# Carba‐*closo*‐dodecaborates as Superchaotropic Anions

**DOI:** 10.1002/chem.71450

**Published:** 2026-07-28

**Authors:** Andrea Barba‐Bon, Guillermo E. Quintero, Maryam Ashjari, Ivana Nikšić‐Franjić, Kai‐Jen Tsung, Xiaojuan Wang, Margarita E. Aliaga, Bohumir Grüner, Werner M. Nau

**Affiliations:** ^1^ School of Science Constructor University Bremen Germany; ^2^ Department of Physical Chemistry Chemistry School Pontificia Universidad Católica De Chile Santiago Chile; ^3^ College of Chemical Engineering China University of Petroleum (East China) Qingdao China; ^4^ Institute of Inorganic Chemistry Czech Academy of Sciences Husinec‐Řež Czech Republic

**Keywords:** Boron clusters, chaotropic effect, cyclodextrins, membrane carrier, superchaotropic ions

## Abstract

The host‐guest chemistry of the 1‐carba‐*closo*‐dodecaborate anion (CB_11_H_12_^–^) and its 12‐monosubstituted derivatives (R = I, Ph, Et, Bu, and OH) with cyclodextrins (CDs) has been studied, a class of weakly coordinating polyhedral boron cluster anions that have already found applications in catalysis and as ionic liquids. These mono‐anions bind surprisingly tightly (*K*_a_  ≈ 10^5^ M^−1^) to the hydrophobic cavity of CDs, particularly the smallest α‐CD and  β‐CD homologues. This binding propensity closely resembles the binding of larger boron clusters, decaborates and dodecaborates (B_10_X_10_^2–^ and B_12_X_12_^2–^, with X = Cl, Br, and I) as well as metallacarboranes (Co(C_2_B_9_H_11_)_2_^–^), which has been attributed to the superchaotropic properties of these large, charge‐dispersed anions. The thermodynamic signature of the encapsulation of 1‐carba‐*closo*‐dodecaborates by CDs is consistent with the chaotropic effect as dominant driving force, that is, the binding is strongly enthalpically driven (Δ*H* down to –19 kcal mol^−1^). The binding of the parent CB_11_H_12_^–^ mono‐anion exceeds the binding of the isosteric B_12_H_12_^2–^ di‐anion to the three CD homologues by 2‐3 orders of magnitude, which indicates a higher chaotropicity, as expected when an ion has a lower charge density. The results position CB_11_H_12_^–^ as the smallest superchaotropic ion known to date, an assignment that was confirmed through its strong effect on the cloud point of a nonionic polymer (hydroxypropylcellulose). The CB_11_H_12_^–^ ion was subsequently also tested as a chaotropic transmembrane carrier, and it was found to be active in delivering hydrophilic peptides (Arg_8_ and phalloidin) through lipid bilayer membranes, in artificial liposomes as well as live CHO‐K1 cells. The binding and transport activities of the different superchaotropic boron cluster anions correlate well with their volume and surface charge density, as descriptors of their chaotropicity.

## Graphical abstract

The singly charged boron cluster anion CB_11_H_12_^–^ forms strong inclusion complexes with cyclodextrins, interacts strongly with nonionic polymers, and serves as an effective transmembrane carrier, positioning itself as the smallest superchaotropic anion known to date.

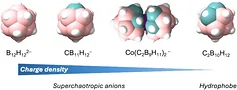

## 1 Introduction

Ten years ago, inorganic boron cluster anions [1, 2, 3, 4], along with polyoxometalates [5, 6, 7], have been found to serve as high‐affinity binders to cyclodextrins (CDs), with affinities approaching the micromolar regime in water [1, 2, 3]. This supramolecular affinity was unexpected, because CDs have been previously recognized as selective binders for hydrophobic organic guest molecules—while the cluster ions carry multiple negative charges. Leaning on early indications for a weak binding of chaotropic ions (e.g. I^–^, SCN^–^, ClO_4_^–^, PF_6_^–^) to CDs [8, 9, 10, 11], it was concluded that the higher binding of the large cluster ions was due to their exceptionally high chaotropicity [1, 12]. Boron clusters and polyoxometalates therefore expand the Hofmeister series of kosmotropic and chaotropic ions; they act as *super*chaotropic ions [13, 14, 15, 16]. In parallel, it was recognized that the affinity of these large, charge‐delocalized cluster ions to hydrophobic cavities and interfaces was a generic feature, extending from macrocycles [1, 2, 3, 4, 5, 6, 7, 17, 18, 19, 20] to peptides and proteins [21, 22, 23, 24, 25], to membranes [26, 27, 28, 29, 30, 31, 32, 33, 34], and to soft matter in general [12, 35, 36, 37, 38]. The affinity relates to an intrinsic and generic driving force for molecular association phenomena that has been described as chaotropic effect [39], complementing the hydrophobic effect as a conceptually new supramolecular assembly motif in water. One key characteristic of the chaotropic effect is that the association is enthalpically driven [1, 7, 39], which is in contrast to association phenomena driven by the classical hydrophobic effect, for which the entropic contribution generally dominates [40, 41, 42].

To explore the full potential of the chaotropic effect and the novel biological and supramolecular properties of superchaotropic anions, it is essential to understand in detail the molecular physicochemical determinants that confer superchaotropic character—empirically manifested through a higher affinity to macrocycles [1, 2, 3, 4, 5, 6, 7], polymers [43, 44, 45], biopolymers [46, 47, 48, 49], proteins [25, 50], and lipid bilayers [26, 27, 28, 29, 30, 31, 32, 33, 34]. Previous work has shown that the superchaotropic character of borate cluster anions increases with their molecular size and associated polarizability [1, 2, 46, 49], for example, when perhalogenated derivatives are compared (B_12_I_12_^2–^ > B_12_Br_12_^2–^ > B_12_Cl_12_^2–^ > B_12_H_12_^2–^) [1, 2, 46] or when two homologues with the same halogens but different size are compared (B_12_X_12_^2–^ > B_10_X_10_^2–^) [49]. The effect of net ionic charge has been addressed for polyoxometalates [36, 51], but not yet for boron clusters, which presents the subject of our present study. Indeed, the icosahedral framework of dodecaborate, B_12_H_12_^2–^, allows the successive replacement of boron atoms by carbon atoms accompanied by a reduction of net charge, yielding first the singly charged 1‐carba‐*closo*‐dodecaborate, CB_11_H_12_^–^ (**1**, also known as monocarborane or carborane anion, Figure 1), and lastly the neutral dicarba‐*closo*‐dodecaborane (carborane) isomers, C_2_B_10_H_12_, all of which being closely comparable in molecular size/volume and polarizability. Carboranes are highly hydrophobic species, which interact through the hydrophobic effect with CDs [52, 53, 54, 55, 56]. Therefore, we focus on the superchaotropic properties of CB_11_H_12_^–^ which has only half the charge density of the isosteric B_12_H_12_^2–^. In addition, this mono‐anion is amenable to substitution chemistry [57], which allows us to further fine‐tune its properties through the substituents in derivatives **2**‐**6**, especially in their binding to CDs. We also tested the potential of CB_11_H_12_^–^ to shift the cloud point of nonionic polymers and in membrane transport assays.

**FIGURE 1:**
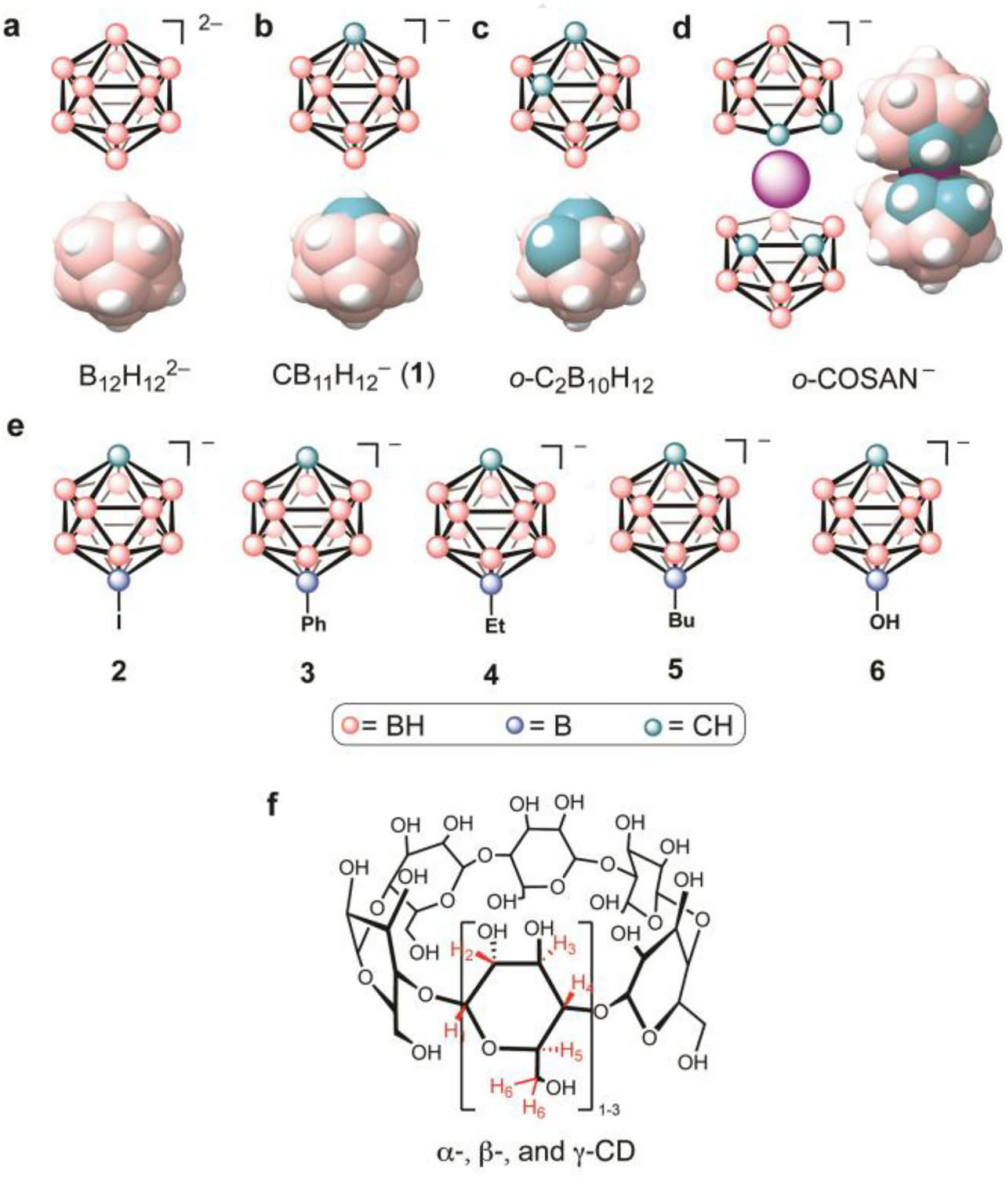
Chemical structures and molecular models of representative boron cluster types: (a) the dodecaborate di‐anion B_12_H_12_^2–^, (b) the 1‐carba‐*closo*‐dodecaborate anion CB_11_H_12_^–^ (**1**), (c) the neutral 1,2‐dicarba‐dodecaborane C_2_B_10_H_12_ (*ortho*‐carborane), and (d) the cobalt bis(dicarbollide) anion (3,3´‐Co(1,2‐C_2_B_9_H_11_)_2_^–^, *o*‐COSAN^–^). (e) Chemical structures of the 1‐carba‐*closo*‐dodecaborate derivatives **2**‐**6** and of α‐, β‐, and γ‐cyclodextrin (f).

## 2 Results

The supramolecular association between the 1‐carba‐*closo*‐dodecaborate anion (**1**, as trimethylammonium salt) [58, 59] and α‐, β‐, and γ‐CD was evaluated first by ^1^H‐NMR spectroscopy (Figures 2a and S1). Complexation‐induced chemical shifts were observed for all three CDs upon cluster addition, pointing to supramolecular complex formation. The most pronounced shifts were observed for α‐CD, strongly downfield for the H3 proton, clearly up‐field for the H1, H2, and H4 protons, and smaller ones for H5 and H6. This pattern is diagnostic for the formation of an inclusion complex, in which the guest molecule protrudes from the wider (upper) rim and fills about half the cavity, but does not reach the bottom region (Figure 1f). In contrast, for γ‐CD, the shift was largest and downfield for the H5 and H6 protons, suggesting a partial inclusion complex of the cluster with the narrower (lower) rim near the primary hydroxyl groups. For β‐CD, sizeable shifts were obtained for both, H3 and H5, indicating a central inclusion of the cluster into the cavity. ^1^H‐NMR titrations were also performed for α‐, β‐, and γ‐CD with all other six cluster derivatives **2**‐**6** (all as trimethylammonium salts, except **3** as cesium salt, Figures S2–S6), and very similar structural trends were observed, except for the hydroxylated derivative (**6**) which showed negligible changes in the ^1^H‐NMR spectra, indicative of weak affinity. It should be noted that the complex between α‐CD and **1** is the only one among the investigated host‐guest complexes that exhibited slow exchange on the ^1^H NMR timescale, consistent with the formation of a tight “capsule”‐like 2:1 complex, later corroborated by ITC.

**FIGURE 2:**
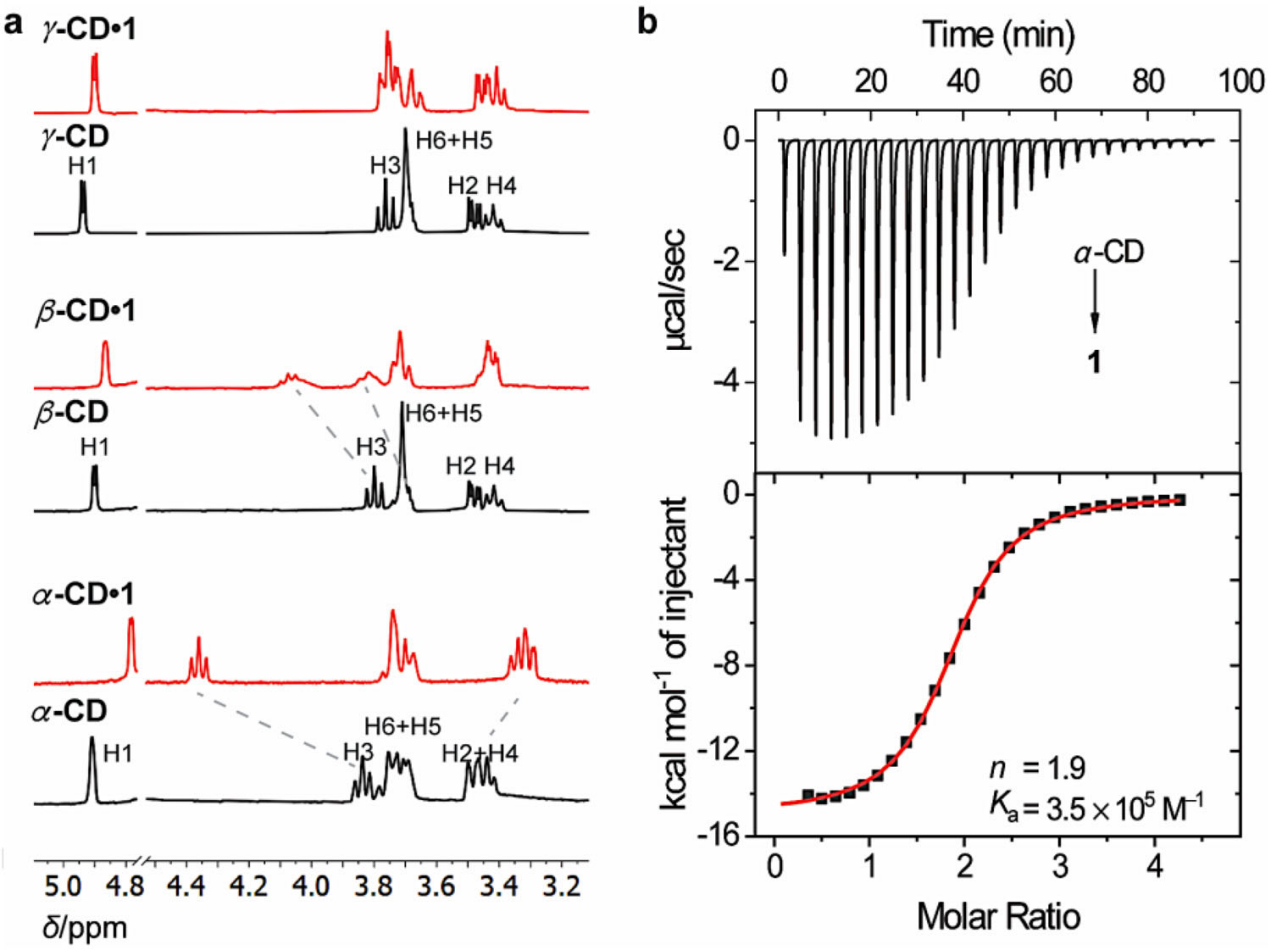
(a) ^1^H NMR spectra of free CDs (500 µM, black) and their complexes with **1** (500 µM, as trimethylammonium salt, red) in D_2_O. (b) Microcalorimetric titration of **1** (50  µM) with α‐CD (1 mM) in water; raw ITC data (top) for sequential injections of cyclodextrin solution into a cluster solution and apparent reaction heats (bottom) obtained from the integration of the calorimetric traces.

The differences in co‐conformations of the host‐guest inclusion complexes observed by ^1^H NMR can be rationalized by the size‐complementarity principle and the approximate dimensions of the CDs [2]. The smallest CD, α‐CD, has rim diameters of ca. 8.0 Å versus 5.0 Å [2] which directs the inclusion of the cluster (ca. 7.6 Å) from the wider rim without allowing a deep immersion. In contrast, the largest CD in this series, γ‐CD, is sufficiently flexible and wide to allow an inclusion near the narrower rim (ca. 7.0 Å), while the upper rim region (10.7 Å) would leave too much free space to optimize dispersion interactions. The cavity of β‐CD appeared to be best suited for full and deep guest immersion, which is confirmed by an almost ideal packing coefficient (*PC*) of 57% (ideal value: 55%) [60], calculated from the volume of the CB_11_H_12_^–^ guest (149 Å^3^) divided by the volume of the β‐CD cavity (262 Å^3^) [61].

To gain more detailed insights into the structures of the supramolecular complexes, we conducted quantum‐chemical (DFT, with dispersion correction) calculations for CB_11_H_12_^–^; the lowest‐energy optimized structures are shown in Figure 3. Different initial geometries were evaluated for each complex, for instance, the relative position of the carbon atom in the cluster vertex was varied and the cluster was placed either near the upper and lower rim of the CDs (Figures S7–S9 and Tables S1–S3). In keeping with the stoichiometry determined by ITC (see below) a 2:1 stoichiometry was used for α‐CD and 1:1 stoichiometries were assumed for β‐CD and γ‐CD. In the ternary host‐guest complex with α‐CD, the cluster anion (**1**) is capped by the wider rim of two α‐CDs, which are held together by intermolecular hydrogen bonds; this is a known complexation pattern for spherical guests encapsulated by CDs [1, 39, 52, 62, 63, 64, 65, 66] and consistent with the NMR assignments. For β‐CD, a central immersion of the guest was found and for the larger γ‐CD the cluster located preferentially near the lower, primary hydroxyl rim, both in conformity with the NMR shift patterns. It should be noted that the principal host‐guest binding mode (2:1 only for α‐CD and 1:1 for the larger CDs) has been previously deduced from structural studies for the isosteric but noncharged *o*‐carborane as guest molecule [52].

**FIGURE 3:**
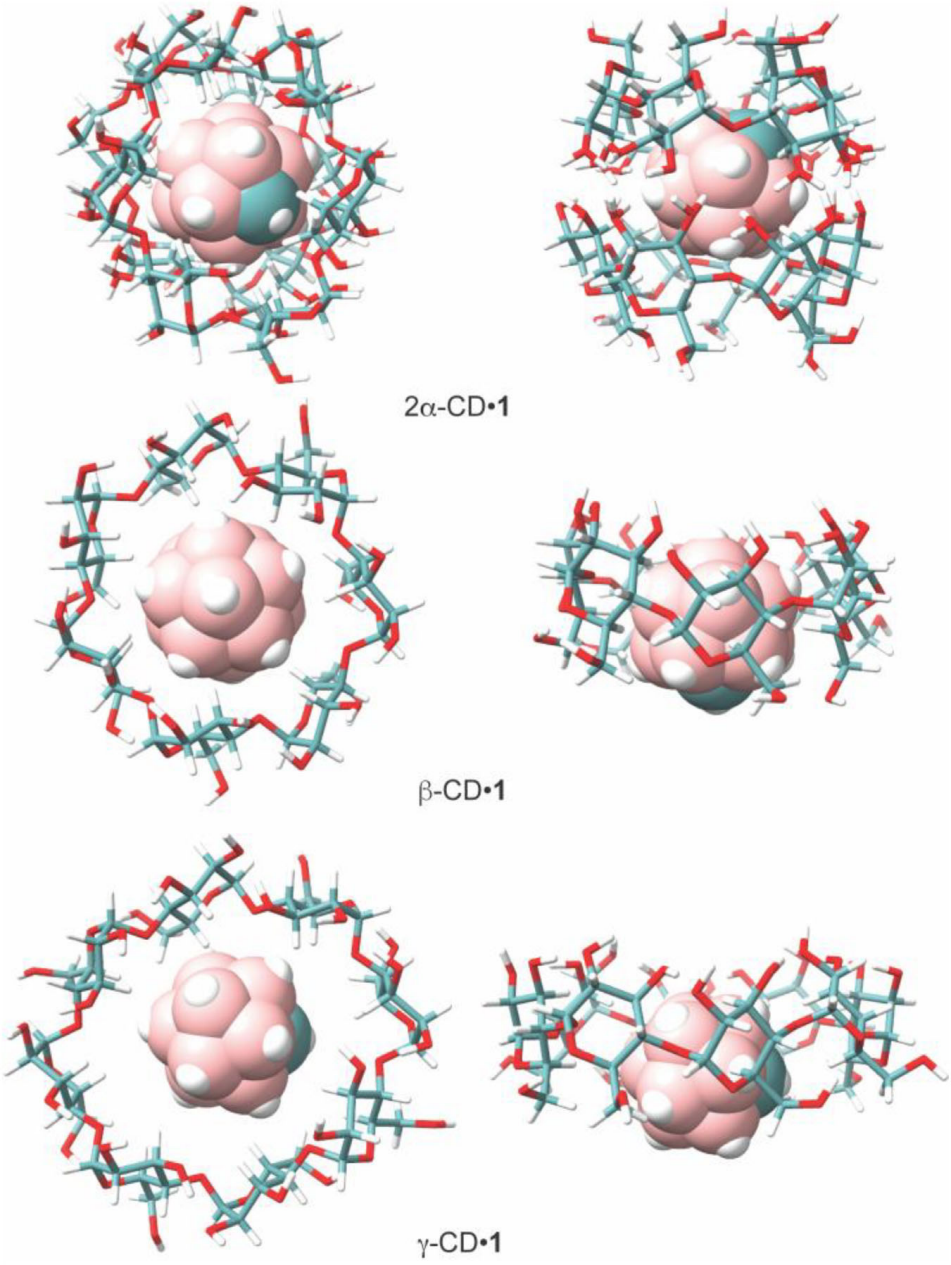
DFT‐optimized structures of the CD•**1** complexes in water (left: top view, right: side view) at the M06‐2X‐D3/3‐21G level of theory (see Methods).

ITC was used to quantify the stoichiometry (*n* value) and thermochemistry (*K*_a_, Δ*H*, Δ*S*) of complexation (Figure 2b; Figures S10–S13; Table 1). The unsubstituted parent cluster (**1**) afforded an *n* value of 1.9, indicative of a 2:1 complexation stoichiometry in which the cluster is surrounded by two α‐CD macrocycles. This was confirmed by the reverse titration (cluster added to α‐CD, see Figure S12), which afforded a stoichiometric value of 0.4 and the same affinity, within error. For the larger CDs, the *n* values were closer to 1, consistent with a stoichiometric 1:1 host‐guest binding.

**TABLE 1:** Binding constants (*K*_a_ in M^−1^) and thermodynamic data (in kcal mol^−1^) measured for the complex formation between α‐CD, β‐CD, and γ‐CD with carba‐*closo*‐dodecaborates **1**−**6** (as trimethylammonium salts, except **3** as cesium salt) in water^a^.

	α‐CD				β‐CD				γ‐CD			
	*n*	*K*_a_ (10^3^)	Δ*H*°	*T*Δ*S*°	*n*	*K*_a_ (10^3^)	Δ*H*°	*T*Δ*S*°	*n*	*K*_a_ (10^3^)	Δ*H*°	*T*Δ*S*°
**1**	1.9	350	−14.9	−7.4	0.9	62	−11.9	−5.4	0.9	230	−7.9	−0.7
**2**		<1^b^			0.9	160	−16.8	−9.7	1.0	640	−9.2	−1.3
**3**		<1^b^			0.9	440	−19.1	−11.4	0.9	790	−9.4	−1.4
**4**		4.4^b^			0.7	130	−16.2	−9.3	0.5	990	−11.7	−3.5
**5**	1.0	3.7	−5.4	−0.6	1.0	610	−15.1	−7.1	0.9	1200	−11.1	−2.8
**6** ^c^	1.0	5.6	− 0.6	4.5		1.4^b^			n.h.^d^			
	1.0	4.0	−23	− 18.1								

^a^ Error data is 10% for *K*_a_ and *n* values and ± 0.5 kcal mol^−1^ for Δ*H*° and *T*Δ*S*° (duplicate measurements).

^b^ From ^1^H‐NMR titrations in D_2_O.

^c^ Data fitted with the “two‐set‐of‐sites” model, which resulted in two binding constants.

^d^ n.h. = no significant binding heat was obtained.

Very high values for the affinity constants, *K*_a_, were obtained, on the order of 10^4^–10^5^ M^−1^ for the parent cluster (**1**), approaching the highest values known for cyclodextrin binding [1, 3, 4, 67]. For example, the binding affinity of **1** with γ‐CD (2.3 × 10^5^ M^−1^) approaches that of 9‐carboxytriamantane (ca. 4 × 10^5^ M^−1^) [67, 68], and that of **1** with β‐CD (6.2 × 10^4^ M^−1^) reaches the value reported for 1‐diamantane carboxylate (ca. 5 × 10^4^ M^−1^) [67, 68]. The binding affinity of **1** with α‐CD stands out the most, because high‐affinity binders for this smallest CD are rare. There are some reports of similarly large binding constants (ca. 3 × 10^5^ M^−1^) for two other chaotropic anions, I_3_^–^ and Fe(CN)_6_^4–^, but these are derived from indirect electrochemical measurements and not from direct titrations [69, 70, 71]. In addition, these two guests are chemically highly reactive (Fe(CN)_6_^4–^) [71] or they exist only in equilibrium (I_3_^–^) [69, 70]. Sodium dodecylsulfate (SDS) [72, 73] is an amphiphilic guest that can also bind two α‐CD macrocycles tightly (*K*_a_ = 2 × 10^4^ M^−1^, taken as square‐root value of the individual binding constants). We confirmed this value by ITC (∼ 3 × 10^4^ M^−1^, Figure S13), about one order of magnitude below the affinity of **1**.

For the antipodally monofunctionalized derivatives **2**‐**6**, the range of binding affinities are comparable to those of **1** for the larger hosts, β‐CD and γ‐CD, that is, on the order of 10^4^–10^5^ M^−1^. The single substituents have a rather small effect on the absolute affinities, likely because they can protrude into one of the CD rim regions without steric interference while retaining the intrinsic strong binding of the boron cluster core. In stark contrast, the high‐affinity binding observed for **1** and α‐CD entirely collapses when a substituent is introduced, as for derivatives **2**‐**5**, which have 2 orders of magnitude or lower binding affinities, albeit shifts in the NMR spectra indicate complexation. Apparently, the stability of the 2:1 complex mainly derives from the full desolvation of the cluster and the full encapsulation, which is feasible for **1**, but not for the substituted derivatives. Unfortunately, the two individual binding affinities (*K*_a1_ for the 1:1 complex formation and *K*_a2_ for the 2:1 complex formation) could not be resolved from the ITC thermogram of **1**, but the low binding measured for **2**‐**5** suggests *K*_a2_>> *K*_a1_.

The ITC data revealed invariably that the complexation of the carba‐*closo‐*dodecaborate derivatives with CDs is enthalpically driven, accompanied by a negative entropic component (Table 1). For the parent compound **1**, the enthalpic effect (normalized per CD) decreases slightly with the size of the macrocycle (from –14.9 to –11.9 to –7.9 kcal mol^−1^), which is diagnostic for a small contribution of high‐energy water release from small host cavities, particularly α‐CD and  β‐CD [8, 41, 74, 75]. The overall thermochemical pattern is diagnostic for supramolecular association phenomena driven by the chaotropic effect [20, 39]. Accordingly, the carba‐*closo‐*dodecaborate ion acts as a chaotropic rather than a hydrophobic anion, and due to the fact that its binding exceeds that of conventional chaotropic anions, such as ClO_4_^–^ and PF_6_^–^, it can also be classified as a superchaotropic anion.

A complementary method to estimate the chaotropicity of anions is to evaluate their affinity to nonionic surfactants [12, 36] or polymers [43, 45, 47], by cloud point measurements. The addition of salts to nonionic surfactants/polymers increases their solubility (salting‐in, chaotropes) or decreases it (salting‐out, kosmotropes). Specifically, we evaluated the effect of different boron cluster anions on hydroxypropylcellulose since it is also composed of glucose units (in analogy to CDs); its native cloud point lies at 47°C [76, 77]. The addition of the boron cluster salts induced a substantial increase of the cloud point (up to 100°C), for the most chaotropic anions even at µM concentrations. In contrast, for the conventional chaotrope ClO_4_^–^ much higher (mM) concentrations were required to achieve small increases in cloud point (Figure 4 and Table 2). This behavior, strong interaction of superchaotropes in  µM concentrations versus weak interaction of conventional chaotropes at mM concentrations, is reminiscent of their interactions with other hydrophobic surfaces and interfaces, for example, lipids [34]. Most importantly, CB_11_H_12_^–^ outperformed the homologous B_12_H_12_^2–^; in the case of the monoanion, ca. 500 µM are sufficient to affect an increase in cloud point by ca. 15°C (an effect which we arbitrarily defined for direct comparison, Table 2) while ca. 100 mM are required for the dianion. Indeed, the effect of the small CB_11_H_12_^–^ was comparable to that of the larger superchaotropic clusters, B_12_Br_12_^2–^ and COSAN^–^.

**FIGURE 4:**
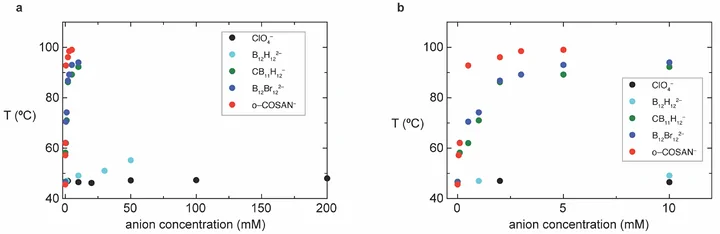
(a) Cloud point of 1% *w*/w hydroxypropylcellulose in water (ca. 26 mM) as a function of increasing anion concentration. (b) Magnified representation of the lower concentration range. Note that at COSAN^–^ concentrations above 6 mM the cloud‐point was no longer accessible (above 100°C).

**TABLE 2:** Anion concentration (*c*_CP_) required to affect an increase in cloud point of a 1% *w*/w hydroxypropylcellulose (ca. 26 mM) by 15°C (from 47°C to 62°C).

Anion	*c*_CP_ (mM)
ClO_4_^–^	>1000
B_12_H_12_^2–^	100
CB_11_H_12_^–^	0.5
B_12_Br_12_^2–^	0.1
*o*‐COSAN^–^	0.1

Another practically appealing feature of superchaotropic anions is their ability to act as transmembrane carriers of impermeable hydrophilic molecules [46, 48, 49, 50, 51, 78, 79, 80, 81, 82]. We therefore studied whether the carba‐*closo*‐dodecaborate anion, despite its small size, would display the same biological activity that has previously only been exhibited by larger boron clusters, namely B_12_Br_12_^2–^ and *o*‐COSAN^–^. COSANs (Figure 1d, from CObalt SANdwich) are metallacarboranes composed of two *nido*‐carborane anions (C_2_B_9_H_11_^2–^) and therefore about double in size, but mono‐anionic, due to the central coordinating Co^3+^ metal ion [3, 48]. The positively charged octaarginine and the neutral peptide phalloidin were used as reference cargo molecules. Experiments with *o*‐C_2_B_10_H_12_ are prevented by its low water solubility. The associated transport studies were conducted in both, model membranes (liposomes, Table 3; Figures S14–S16; Tables S6 and S7) and CHO‐K1 cells (Table 3; Figure 5; Figures S17–S19). The combined results showed that CB_11_H_12_^–^ is a potent transmembrane carrier of both peptides, which is in line with its superchaotropic behavior. However, it also showed cellular toxicity at high concentrations (>100 µM); this is expected for all superchaotropic anions, because they interact with lipid bilayer membranes [26, 27, 34] and show, among others, concentration‐dependent membrane‐lytic effects [31]. The transport experiments do therefore need to be performed in the activity range around the *EC*_50_ values (ca. 50 µM for **1**, see Table S6) but below the *IC*_50_ value (ca. 200 µM for **1**, Table 3).

**TABLE 3:** Membrane transport efficiency (*E*_a_) of different boron clusters in model membranes with octaarginine and phalloidin as cargo, their cellular uptake efficiency with TAMRA‐Arg_8_ as cargo, and toxicity (as *IC*_50_ value).

Clusters	Vesicles (*E*_a_)^b^		Cells^c^	
	Octaarginine	Phalloidin	Cellular uptake (%)^d^	*IC*_50_ ( µM)^e^
CB_11_H_12_^–^ (**1**)^a^	5.1	4.8	60	200
*o*‐COSAN^–^	12	No activity	81	7.5
B_12_H_12_^2–^	No activity	No activity	<5	>5000
B_12_Br_12_^2–^	6.1	4.3	61	260

^a^ Used as N(CH_3_)_3_H^+^ salt; analogous experiments with the sodium salt confirmed that the counter cation does not significantly affect the transport activity (Figure S16 and Table S7).

^b^ Membrane transport efficiency calculated in EYPC vesicles, see Supporting Information for details. Error in data is 10% (SD).

^c^ CHO‐K1 cell line.

^d^ By flow cytometry, calculated as % increase in relation to the incubation with TAMRA‐Arg_8_ alone. Error in data is 10%.

^e^ Calculated by resazurin assay, see Methods for details. Error in data is 10% (SD).

**FIGURE 5:**
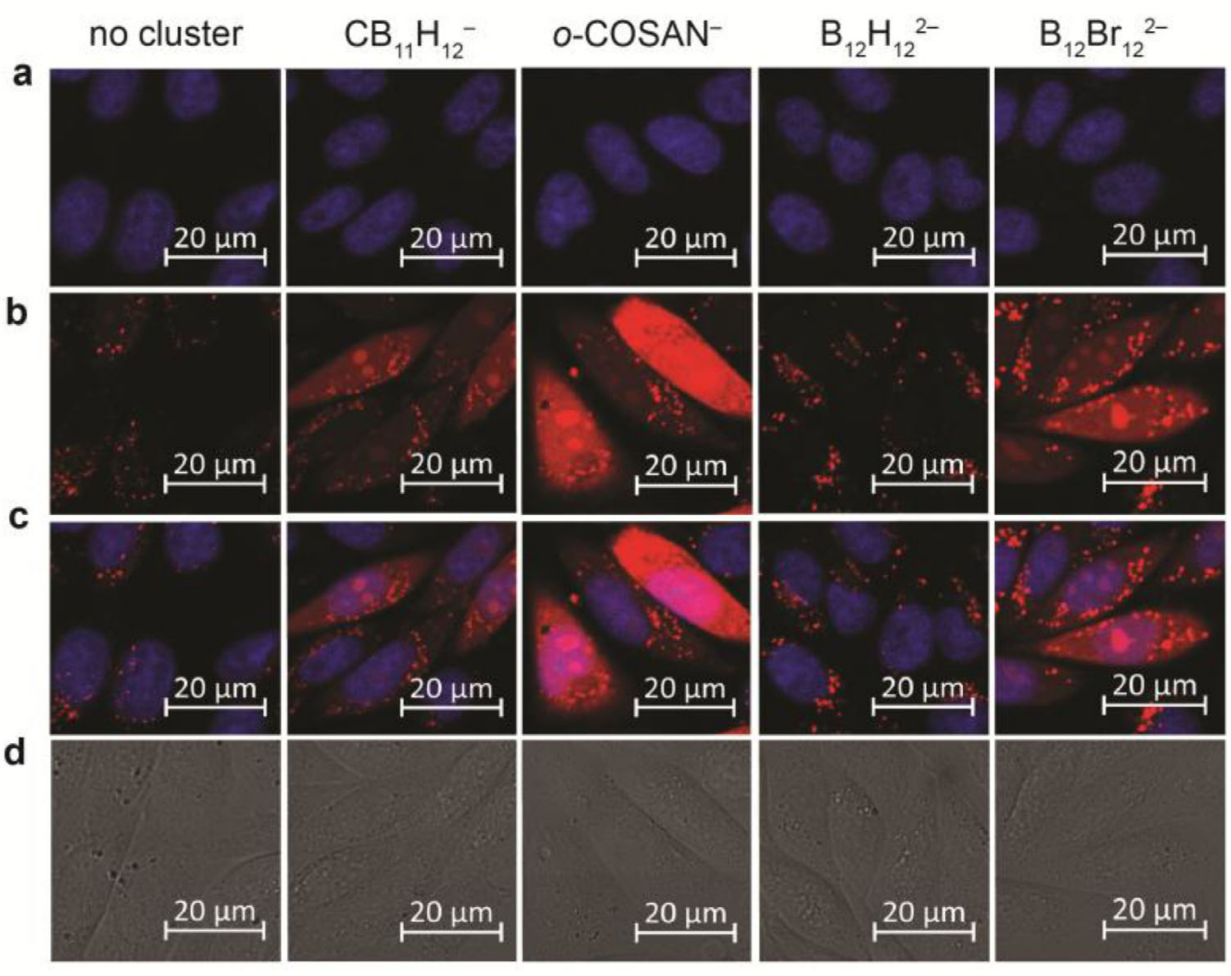
Confocal images of live CHO‐K1 cells incubated with 5 µM TAMRA‐Arg_8_ alone (no carrier) and in the presence of boron clusters: CB_11_H_12_^−^, *o*‐COSAN^–^, B_12_H_12_^2−^, and B_12_Br_12_^2−^ (5 µM). (a) Hoechst channel (nucleus); (b) TAMRA channel; (c) merged channels; and (d) bright field.

To gain mechanistic insight, the intracellular delivery was also evaluated in the presence of endocytosis inhibitors (dynasore [83] and chlorpromazine [84]) by confocal microscopy (Figures S18,S19). The cellular uptake was not significantly affected by the two inhibitors, pointing to a dominant energy‐independent internalization mechanism (e.g. lipid bilayer permeation) rather than endocytotic pathways, as previously observed for other boron cluster carriers [46, 48].

## 3 Discussion

Boron clusters are man‐made compounds, in which the framework can be crafted by boron atoms only (dodecaborate di‐anions, e.g. B_12_H_12_^2–^) or in combination with one or two carbon atoms (carba‐*closo*‐dodecaborate anions, e.g. CB_11_H_12_^–^ (**1**) and carboranes, e.g. C_2_B_10_H_12_, Figure 1). The three closed (“*closo*”) clusters have the same icosahedral and almost globular geometry, virtually identical size, and they are stabilized by a special form of *σ* bond‐delocalized 3D‐aromaticity [85, 86, 87]. They are jointly characterized by being chemically and thermally highly resistant [86, 87, 88] as well as inert toward biological degradation [88]. Indeed, all three have already been considered from quite different application perspectives; for example, dodecaborates have received attention in the context of boron neutron capture therapy [26, 89, 90, 91, 92], carba‐*closo*‐dodecaborates as ionic liquids and battery electrolytes [93, 94, 95, 96, 97], and carboranes as pharmacophores [88, 98, 99, 100]. Despite their structural similarities, the hydration properties of the dodecaborate anion (B_12_H_12_^2–^) and carboranes (C_2_B_10_H_12_) are very different: the former acts as a superchaotrope [1, 46] and the latter as a hydrophobe [99]. Our present study reveals that the “missing link” of the globular clusters, the mono‐anionic carba‐*closo*‐dodecaborate **1**, behaves as an even more powerful superchaotrope than B_12_H_12_^2–^.

The carba‐*closo*‐dodecaborate **1** and some of its derivatives show very high affinities to α‐CD and  β‐CD, up to 10^5^ M^−1^. Thousands of guest molecules are known that bind to these two smallest CDs with affinities on the order of 10^3^ M^−1^ or lower [8, 9, 10, 11, 20, 75, 101, 102, 103, 104, 105], which makes these high affinities of an inorganic anion unexpected and special. A similar surprise was found ten years ago when the halogenated dodecaborate clusters (B_12_X_12_^2–^, X = Cl, Br, and I) emerged as high‐affinity binders (10^5^‐10^6^ M^−1^) for the larger cyclodextrins (γ‐CD, δ‐CD, ε‐CD, and ζ‐CD) [1, 2, 66], rivaling the best known (organic) guest molecules for γ‐CD known by then [68, 106, 107], and in some cases (for δ‐CD) outperforming reported binding constants by up to 6 orders of magnitude [1, 66]. In all cases, and also for the carba‐*closo*‐dodecaborate **1**, this binding can be attributed to the superchaotropic nature of the cluster ions and the chaotropic effect as driving force, manifested in highly negative complexation enthalpies. It is also intriguing that CB_11_H_12_^–^ (**1**) acts as a rather promiscuous high‐affinity binder to all three CD homologues, α‐CD,  β‐CD, and γ‐CD; this can be rationalized from the NMR spectra and computed structures (Figures 2 and 3), which show that the cluster has an ideal size to form inclusion complexes with all three CDs, albeit in different regions (upper rim for  α‐CD, center for  β‐CD, and lower rim for γ‐CD).

The molecular sizes/volumes (149±2 Å^3^) and polarizabilities (22±4 Å^3^) of the three related boron clusters B_12_H_12_^2–^, CB_11_H_12_^–^ (**1**), and C_2_B_10_H_12_ are essentially the same (Figure 1a–c and Table 4). However, their binding affinities to  β‐CD, as a common host, show a peculiar up and down trend, namely, quite weak for B_12_H_12_^2–^ (*K*_a_ = 200 M^−1^), [1] very strong for CB_11_H_12_^–^ (**1**, ca. 10^5^ M^−1^), and weaker again for C_2_B_10_H_12_ (ca. 10^3^ M^−1^) [54, 55, 56]. This applies also to their monosubstituted derivatives [1, 55]. These variations can now be rationalized. Carboranes (C_2_B_10_H_12_) are hydrophobic, poorly water‐soluble species, frequently compared to adamantanes, such that their binding can become entropically driven [55], especially for larger macrocycles such as γ‐CD where the release of high‐energy water does not contribute [1, 41, 67, 75, 101]. B_12_H_12_^2–^ and CB_11_H_12_^–^ are both superchaotropic species, but the charge of CB_11_H_12_^–^ is about half, resulting in a higher chaotropicity and the observed very strong binding to CDs. In fact, the binding affinity of **1** is comparable to the association constants of the larger superchaotropic di‐anion B_12_Br_12_^2–^ to γ‐CD (9.5 × 10^5^ M^−1^) [1] and the larger double‐cluster mono‐anion COSAN^–^ to γ‐CD (1.9 × 10^5^ M^−1^) [3], see Figure 1. Since the binding of anions to CDs cannot be used as a rigorous chaotropicity scale, because size and shape matching between the guest and the cavity are also required, we performed cloud‐point measurements with nonionic polymers (Figure 4 and Table 2). Since the interaction of anions with polymers is not subject to the same structural requirements for cyclodextrin binding, shifts of the cloud points have been considered as alternative measure of chaotropicity [12, 36, 43, 45, 47]. Indeed, also in these experiments, CB_11_H_12_^–^ shifted the cloud point more strongly than B_12_H_12_^2–^ and the effect of CB_11_H_12_^–^ was comparable to that of the larger superchaotropic clusters, B_12_Br_12_^2–^ and COSAN^–^, corroborating the classification of CB_11_H_12_^–^ as a superchaotropic anion.

**TABLE 4:** Molecular properties of selected boron clusters. Charge (*q*), dipole moment (*μ*), polarizability (*α*), molecular volume (*V*), surface area (*A*), volume charge density (*ρ*), and surface charge density (*σ*).

Clusters	*q* (–*e*)	*μ* (D)^a^	*α* (Å^3^)^a^	*V* (Å^3^)^a^ ^,^ ^b^	*A* (Å^2^)^a^ ^,^ ^b^	*ρ* (–10^−3^ *e*Å^−3^)^c^	*σ* (–10^−3^ *e*Å^−2^)^d^
B_12_H_12_^2–^	2	0	25.1	151	174	13.2	11.5
CB_11_H_12_^–^	1	2.7	21.1	149	170	6.7	5.9
*o*‐C_2_B_10_H_12_	0	4.2	18.6	147	167	0	0
B_12_Br_12_^2–^	2	0	60.2	423	473	4.7	4.2
*o*‐COSAN^–^	1	0–6^e^	42.9	281	302	3.6	3.3

^a^ From DFT‐calculated gas‐phase structures (B3LYP‐D3/6‐311+G(2d,p) level of theory, LANL2DZdp ECP basis set for cobalt).

^b^ Molecular volumes and surface areas calculated as the respective van‐der‐Waals values in HyperChem software (see Methods in Supporting Information); surface area obtained by the “grid” method.

^c^ Volume charge density was calculated as *ρ = q*/*V*.

^d^ Surface charge density was calculated as *σ = q*/*A*.

^e^ Rotamer‐dependent dipole moment.

The combined results demonstrate that the chaotropicity of boron cluster anions can be modulated in several ways: by lowering their charge (CB_11_H_12_^–^ > B_12_H_12_^2–^), by increasing their size (B_12_Br_12_^2–^ > B_12_H_12_^2–^), or both (COSAN^–^ >> B_12_H_12_^2–^), as schematically summarized in Figure 6. A correlation of cluster size/polarizability of anions with their chaotropicity at identical negative charge (–2) has been previously documented for the decaborate and dodecaborate series (B_10_X_10_^2–^ and B_12_X_12_^2–^, X = H, Cl, Br, and I) [15, 49]. Conversely, an inverse correlation of net charge of an inorganic ion with its chaotropicity has been shown before for polyoxometalates, which are another class of superchaotropic anions whose charge can be varied between –3 and –5 without varying their molecular size significantly [7, 36, 45, 51].

**FIGURE 6:**
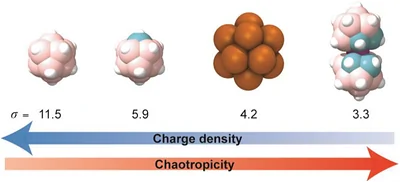
Trends of chaotropicity variation of the four boron cluster anions from Figure 1a–d scaled with decreasing surface charge density (values for *σ*, in units –10^3^ *e*Å^−2^, from Table 4).

Previous studies on boron cluster chaotropicity were not suited to differentiate between the trends of size versus polarizability [49], because the two molecular properties are generally correlated [108, 109]. Interestingly, in the series B_12_H_12_^2–^, CB_11_H_12_^–^, and C_2_B_10_H_12_, the size contraction is vanishingly small (from 151 to 147 Å^3^), but the polarizability of the clusters drops slightly (from 25.1 to 18.6 Å^3^), due to the stepwise removal of two delocalized electrons and the increased dipolar or quadrupolar character of the three clusters (Table 4). Therefore, while higher polarizability of the clusters must play an important role in increasing the stability of the resulting host‐guest complexes on account of improved dispersion interactions [109], it is not dominant in this homologous series, because it would predict the highest affinity for the most polarizable cluster (B_12_H_12_^2–^), which is not observed. Consequently, hydration and dehydration effects, which are the second denominator of the chaotropic effect, must play the essential role, and these are primarily dominated by the net charge (or charge density) when comparing clusters of the same size [7, 36, 51]. This aligns with the view that it is mainly the size and charge of the solute (the charge density) which determine its superchaotropic and hydrophobic character [110], while dispersion interactions may play an enhancing role.

Indeed, the calculated volume as well as surface charge densities, *ρ* and *σ* (Table 4), follow the order B_12_H_12_^2–^ > CB_11_H_12_^–^ > B_12_Br_12_^2–^ > COSAN^–^, and the chaotropicity of the ions is expected to rise in this order. This is consistent with the large majority of experimental results, including their affinity to peptides, membranes [34, 49], and nonionic polymers. COSANs show strong superchaotropic properties in many experiments [3, 20, 25, 48] but displays also an onset of ionic hydrophobic or amphiphilic properties such as self‐assembly [111, 112, 113, 114]. This transitioning from superchaotropic to hydrophobic ionic properties fully aligns with the continuous scale for aqueous solvation [39]. At one point, when the charge of the anion becomes too dilute and when the anion becomes too large, one expects this shift from superchaotropic to hydrophobic ionic properties, which eventually marks the transition to true hydrophobes with vanishing charge density, such as *o*‐carborane, which falls out of the charge density correlation in Table 4.

Besides these fundamental and conceptual advancements, research on new superchaotropic anions in general and their use as new high‐affinity binders to CDs bears practical relevance. Superchaotropic anions have found, during the past four years, biological applications not only in transmembrane transport for drug delivery and cellular staining [46, 48, 49, 50, 51, 78, 79, 80, 81] but also for the stabilization of proteins [115, 116]. Superchaotropic clusters are nowadays also used as templates in the synthesis of large‐ring CDs [66, 73] and, in combination with CDs, as X‐ray contrast agents (B_12_I_12_^2–^) [65]. Since CB_11_H_12_^–^ binds tightly to all three common CDs, its use as a template appears less suitable, but its high chaotropicity implies high potential as transmembrane transporter. Indeed, the transport activity was found to be comparable to the larger brominated B_12_Br_12_^2–^ cluster, outperforming the homologous hydrogenated B_12_H_12_^2–^ cluster, which itself shows only weak activity as transmembrane carrier (Figures S11 and S12; Table 3) [46, 49]. For the transport of octaarginine, the activity of the prototypal boron clusters can be ranked as *o*‐COSAN^–^ > B_12_Br_12_^2–^  ≈ CB_11_H_12_^–^ >> B_12_H_12_^2–^, which is again in line with the trends in charge densities. In addition, its cargo selectivity was also the same as for B_12_Br_12_^2–^, that is, CB_11_H_12_^–^ was also able to transport phalloidin, while the larger COSAN^–^ cluster does not transport this neutral peptide. Consequently, the carba‐*closo*‐dodecaborate anion acts as a highly effective transmembrane carrier of hydrophilic peptides, and it constitutes the smallest anion known to date with this distinct biological activity. In particular, the (smaller) decaborate cluster anion, B_10_H_10_^2–^, does not affect membrane transport of peptides [49].

## 4 Conclusions

Carba‐*closo*‐dodecaborate anions form strong inclusion complexes with CDs. ^1^H‐NMR spectroscopy and quantum‐chemical calculations pointed to a preferred 2:1 host‐guest complexation pattern for the smaller α‐CD and a 1:1 binding mode for the larger CDs. ITC experiments were used to determine the affinity constants (ca. 10^5^ M^−1^) and the thermodynamics of the association. The enthalpically driven binding of CB_11_H_12_^–^ to CDs is diagnostic for the chaotropic effect and allows the classification of this anion as a (super)chaotrope. This assignment was validated by cloud‐point measurements, which showed comparable effects as for other superchaotropic anions (B_12_Br_12_^2–^and *o*‐COSAN^–^). Subsequently, we successfully investigated its use as a molecular transmembrane carrier for otherwise impermeable peptides, establishing CB_11_H_12_^–^ as the first hydrogenated *closo*‐borate cluster anion that is highly active in membrane transport. Comparison of the binding of carba‐*closo*‐dodecaborate to the hydrophobic CD cavities and its transport activity through membranes with the isosteric di‐anionic cluster (B_12_H_12_^2–^) and the mono‐anionic but about double‐sized cluster (*o*‐COSAN^–^) revealed that the chaotropicity is inversely dependent on charge density. It transpires that higher chaotropicity of a boron cluster anion can not only be achieved by choosing a larger molecular volume as previously anticipated (e.g., by employing B_12_Br_12_^2–^ or COSANs instead of B_12_H_12_^2–^) but the same absolute effect can be achieved by reducing the net charge of the anion without altering its size (by employing CB_11_H_12_^–^ instead of B_12_H_12_^2–^).

## Conflicts of Interest

The authors declare no conflicts of interest.

## Supporting information



The authors have cited additional references within the Supporting Information [1–18].
**Supporting File 1**: chem71450‐sup‐0001‐SuppMat.pdf.
