# Supplementary material for: Carba‐closo‐dodecaborates as Superchaotropic Anions
Source: Chemistry. 2026 Jul 28;32(40):e71450. doi: 10.1002/chem.71450 (PMC13716010; doi:10.1002/chem.71450)
Supplement: Supplementary file 1 [file CHEM-32-e71450-s001.pdf]

Supporting information:

Carba-*closo*-dodecaborates as Superchaotropic Anions

Andrea Barba-Bon*[a] Guillermo E. Quintero,[a],[b] Maryam Ashjari,[a] Ivana Nikšić-Franjić,[a] Kai-Jen Tsung,[a] Xiaojuan Wang,[c] Margarita E. Aliaga,[b] Bohumir Grüner,[d] and Werner M. Nau*[a]

[a] Dr. A.Barba-Bon, G. E. Quintero, M. Ashjari, Dr.I. Nikšić-Franjić, K.-J. Tsung, Prof. Dr. W. M. Nau
School of Science
Constructor University
D-28759 Bremen, Germany
E-mail: abarbabon@constructor.university, w.nau@constructor.university

[b] G. E. Quintero, Prof. M. E. Aliaga
Department of Physical Chemistry, Chemistry School
Pontificia Universidad Católica de Chile
8320165 Santiago, Chile

[c] Prof. Dr. X. Wang
College of Chemical Engineering,
China University of Petroleum (East China)
Qingdao 266580, China

[d] Prof. Dr. B. Grüner
Institute of Inorganic Chemistry
Czech Academy of Sciences
CZ-250 68 Řež, Czech Republic

Table of Contents

1. *Materials* ... *2*
2. *Nuclear magnetic resonance (NMR) and mass spectrometry* ... *2*
3. *Isothermal titration calorimetry (ITC)* ... *2*
4. *Vesicle experiments* ... *2*
5. *Cellular uptake* ... *3*
6. *Cellular viability* ... *4*
7. *Computational details* ... *4*
8. *Cloud-point experiments* ... *4*
9. *Results* ... *5*
   - 9.1. *NMR* ... *5*
   - 9.2. *Molecular Dynamic simulations* ... *7*
   - 9.3. *Isothermal titration calorimetry (ITC)* ... *11*
   - 9.4. *Transport experiments in vesicles* ... *14*
   - 9.5. *Cellular uptake* ... *16*
   - 9.6. *Cellular viability* ... *17*
10. *References* ... *18*

1. Materials

Carba-*closo*-dodecaborate anions (**1**-**6**) were synthesized as trimethylammonium salts (except **3**, which was used as cesium salt) as previously reported.[1-4] Sample identity and purity was established by their NMR spectra (which were idetical to the pubished ones) and their precise molecular masses, established by high-resolution mass spectrometry, see details below. The solubility of these derivatives in water was evaluated up 0.5 mM (compound **1** up to 2 mM), which was sufficient for the conducted experiments. Up to this concentration, no nanoaggregates were detected in dynamic light scattering exeriments. The sodium salt of 1-carba-*closo*-dodecaborate (**1**) was also purchased from Katchem (Prague, Czech Republic) for control experiments. α-,β-,γ-Cyclodextrins were purchased from CycloLab Ltd. and used without further purification (corrections for 10-25% water content in the commercial samples was made). Bisbenzimide H 33342 trihydrochloride (Hoechst 33342), egg yolk phosphatidylcholine (EYPC), hydroxypropylcellulose, phalloidin, *p*-xylol-bis-(N-pyridiniumbromide) (DPX), trisodium 8-hydroxypyrene-1,3,6-trisulfonate (HPTS), and Triton X-100 (TX-100) were from Sigma-Aldrich (Steinheim, Germany). CHO-K1 cells were obtained from Hölzel (Cologne, Germany). Dulbecco's Phosphate-Buffered Saline (PBS), penicillin-streptomycin, and trypsin-EDTA (0.25%) were from ThermoFisher (Massachusetts, USA). Ham's F-12 Medium with L-glutamine, Hanks' Balanced Salt Solution (HBSS), and fetal bovine serum (FBS) were from Capricorn Scientific (Ebsdorfergrund, Germany). Ham's F-12 without phenol-red was from Bio-techne (Minneapolis, USA). Heparin and resazurin were from TCI (Eschborn, Germany). Tryptophan-octaarginine (Trp-Arg_8) and TAMRA-octaarginine (TAMRA-Arg_8) were custom-made by GeneCust (Boynes, France) in >98% purity, confirmed by HPLC and MS by the supplier. Peptide stock solution was prepared in water and the concentration was determined by using the extinction coefficient of tryptophan at 280 nm (ε = 5540 $cm^{-1}M^{-1}$)[5] or TAMRA at 578 nm (ε = 90000 $cm^{-1}M^{-1}$).[6]

<u>*Carba-closo-dodecaborate anions characterization:*</u>

1: for Me_3NH^+ salt: ^{1}H NMR (acetone-d_6, 400 MHz, 298K) δ: 3.22 [s, 9H, NMe_3^+], 2.20 [s, 2H, CH, B(12)], 1.44 [m, 10H, B(7-11)]. ^{11}B NMR (acetone-d_6, 128MHz, 298K) δ: –7.96 [d, 1B, BH(12)], –14.13 [d, 5B, BH(7-12)], –17.19 [d, 5B, BH(2-6)]. ^{11}B{1H} NMR (acetone-d_6, 128 MHz, 298K) δ: –7.72 [s, 1B, B(12)], –14.13 [s, 5B, B(7-12)], –17.19 [s, 5B, B(2-6)]. HRMS (ESI-TOF) m/z Calc. for $[CB_{11}H_{12}]^-$: 143.1958; Found: 143.2153.

2: for Me_3NH^+ salt:^{1}H NMR (acetone-d_6, 400 MHz, 298K) δ: 3.22 [s, 9H, NMe_3^+], 2.54 [s, 1H, CH], 2.05 [m, 10B(7-11)]. ^{11}B NMR (acetone-d_6, 128 MHz, 298K) δ:–12.06 [d, 5B, BH(7-12)], –17.71 [m, 6B, BH(2-6), B(12)]. ^{11}B{1H} NMR (acetone-d_6, 128 MHz, 298K) δ:–12.71 [s, 5B, B(7-12)], –17.15 [s, 5B, B(2-6)] –18.79 [s, 1B, B(12)],. HRMS (ESI-TOF) m/z Calc. for $[I\text{-}CB_{11}H_{11}]^-$: 269.0924; Found: 269.1270.

3: for Cs^+ salt: ^{1}H NMR (acetone-d_6, 400 MHz, 298K) δ: 7.39 [d, 2H, BC_6H_5], 6.97 [t, 2H, BC_6H_5], 6.90 [t, 1H, BC_6H_5], 2.22 [s, 1H, CH], 1.75 [m, 10H, B(2-11)]. ^{11}B NMR (acetone-d_6, 128 MHz, 298K) δ: 1.76 [s, 1B, BH(12)], –13.47 [d, 5B, BH(7-12)], –17.52 [d, 5B, BH(2-6)]. ^{11}B{1H} NMR (acetone-d_6, 128 MHz, 298K) δ: 1.64 [s, 1B, B(12)], –13.45 [s, 5B, B(7-12)], –17.57 [s, 5B, B(2-6)]. HRMS (ESI-TOF) m/z Calc. for $[Ph\text{-}CB_{11}H_{11z}]^-$: 219.2274; Found: 219,2545.

4: for Me_4N^+ salt: ^{1}H NMR (acetone-d_6, 400 MHz, 298K) δ: 3.44 [s, 12H, NMe_4^+], 2.17 [s, 1H, CH], 1.41 [m, 10H, B(7-11), 0.76 [s, 3H, CH_3], 0.51 [s, 2H, BCH_2]. ^{11}B NMR (acetone-d_6, 128 MHz, 298K) δ: 3.01 [s, 1B, B(12)], –13.69 [d, 5B, BH(7-12)], –17.85 [d, 5B, BH(2-6)]. ^{11}B{1H} NMR (acetone-d_6, 128 MHz, 298K) δ: 3.03 [s, 1B, B(12)], –13.66 [s, 5B, B (7-12)], –17.83 [s, 5B, B (2-6)]. HRMS (ESI-TOF) m/z Calc. for $[Et\text{-}CB_{11}H_{11}]^-$: 171.2272; Found: 171.2547.

5: for Me_4N^+ salt: ^{1}H NMR (acetone-d_6, 400 MHz, 298K) δ: 3.45 [s, 12H, NMe_4^+], 2.17 [s, 1H, CH], 1.44 [m, H, B(7-11)], 1.19 [m, 4H, CH_2], 0.79 [m, 3H, CH_3], 0.52 [s, 2H, BCH_2]. ^{11}B NMR (acetone-d_6, 128 MHz, 298K) δ: 2.41 [s, 1B, B(12)], –13.55 [d, 5B, BH(7-12)], –17.85 [d, 5B, BH(2-6)]. ^{11}B{1H} NMR (acetone-d_6, 128 MHz, 298K) δ: 2.42 [s, 1B, B(12)], –13.58 [s, 5B, B(7-12)], –17.88 [s, 5B, B(2-6)]. HRMS (ESI-TOF) m/z Calc. for $[Bu\text{-}CB_{11}H_{11}]^-$: 199.2586; Found: 199.2901.

6: for Me_3NH^+ salt ^{1}H NMR (acetone-d_6, 400 MHz, 298K) δ: very broad peaks.^{11}B NMR (acetone-d_6, 128 MHz, 298K) δ: 9.84 [s, 1B, B(12)], –15.11 [d, 5B, BH(7-12)], –19.70 [d, 5B, BH(2-6)]. ^{11}B{1H} NMR (acetone-d_6, 128 MHz, 298K) δ: 9.83 [s, 1B, B (12)], –15.11 [s, 5B, B(7-12)], –19.70 [s, 5B, B(2-6)]. HRMS (ESI-TOF) m/z Calc. for $[OH\text{-}CB_{11}H_{11}]^-$: 159.1907; Found: 159.2186.

2. Nuclear magnetic resonance (NMR) and mass spectrometry

NMR spectra were measured in D_2O at 20 ºC on a JEOL 400MHz spectrometer. The 1D ^{1}H spectra were recorded with single pulse sequence at 90º degree angle, 2 pre-scans, 64 scans and 5s relaxion decay. The 1D ^{11}B spectra were recorded with a single pulse sequence at 90º degree angle, 2 pre-scans, 32 scans, and a 2s relaxion decay. Spectra were analyzed with MestreNova14. Mass spectra were recorded on a Waters QTOF Premier spectrometer. Spectra were analyzed with MassLynx V4.2.

3. Isothermal titration calorimetry (ITC)

All experiments were performed in a VP-ITC MicroCalorimeter from MicroCal in water at atmospheric pressure and 25 ºC. Solutions were degassed and thermostated prior to the titration experiments in a ThermoVac accessory. A constant volume of cyclodextrin solution (10 µL per injection) was titrated into the carba-*closo*-dodecaborate cluster solution, or *vice versa*. Dilution heats were determined by titration of cyclodextrin into water and subtracted from the reaction heat. The neat reaction heat was fitted with Origin 7.0 software by using a "*one-set-of-sites"* or *"two-set-of-sites"* model to obtain the complex stability constant (K_a) and molar reaction enthalpy (ΔH^o). The free energy (ΔG^o) and entropy changes (ΔS^o) were obtained according to $\Delta G^o = -RT\ln K_a = \Delta H^o - T\Delta S^o$.

4. Vesicle experiments

<u>*Vesicle preparation:*</u> A thin lipid film was prepared by evaporating a solution of EYPC in $CHCl_3$ (25 mg/ml) with a stream of nitrogen and then dried *in vacuo* overnight. The resulting film was hydrated with 1 mL buffer (5 mM HPTS, 16.5 mM DPX, 10 mM Tris, 72 mM NaCl, pH 7.4) for 30 min at room temperature, then subjected to freeze–thaw cycles (7 times) and extrusion (15 times) through a polycarbonate membrane (pore

size 100 nm). Extravesicular components were removed by size exclusion chromatography (NAP-25 column) with 10 mM Tris and 107 mM NaCl at pH 7.4. Phospholipid concentrations were calculated by the Stewart assay.[7] The EYPC⊃CF liposomes were prepared analogously, except for the type of the rehydration/elution buffer, which were 50 mM CF, 10 mM Hepes or 10 mM Hepes and 107 mM NaCl, both at pH 7.5.

Transport experiments: EYPC⊃HPTS/DPX liposomes were diluted with buffer (10 mM Tris, 107 mM NaCl, pH 7.4) in a disposable plastic cuvette and gently stirred (total volume 2,000 µL, final lipid concentration 13 µM). HPTS fluorescence was monitored at λ_{em} = 511 nm (λ_{ex} = 413 nm) as a function of time after addition of the boron cluster carrier at 50 s, the cargo peptide at 100 s, and Triton X-100 (30 µL 1.2% (wt/vol)) at 600 s to lyse the vesicles, for calibration. Fluorescence intensities were normalized to fractional emission intensity *I(t)* using equation 1:

$$I(t) = \frac{I_t - I_0}{I_\infty - I_0} \quad (1)$$

where $I_0 = I_t$ before cluster addition and $I_\infty = I_t$ after lysis.
For Hill analysis, I_t before lysis was defined as transmembrane activity, Y, plotted against cluster concentration, c, and fitted to the Hill equation 2 to afford the transmembrane activity in the absence of carrier, Y_0, the maximal transmembrane activity, Y_{max}, the concentration needed to achieve 50% of maximal activity, EC_{50}, and the Hill coefficient, n.

$$Y = Y_0 + \frac{Y_{max} - Y_0}{1 + \left(\frac{EC_{50}}{c}\right)^n} \quad (2)$$

Transport experiments with EYPC⊃CF liposomes were conducted similarly with the corresponding buffer (10 mM Hepes, 107 mM NaCl, pH 7.5) and fluorescence was measured at the optimal parameters for CF, namely λ_{ex}= 492 nm and λ_{em} = 517 nm. A schematic representation of the fluorescence assay principle is shown in Scheme S1.

Activator efficiency: The activator efficiency, E, denotes the ability of a carrier to facilitate the transport of the impermeable cargo and it is characterized by its maximal activity, Y_{max}, as well as the concentration needed to achieve 50% of its maximal activity, EC_{50}. The "best" activator would be one that reaches high Y_{max} at low EC_{50}. To account for both parameters, the activator efficiency is defined as

$$E = Y_{max} \frac{pEC_{50}}{f} \quad (3)$$

where pEC$_{50}$ is the negative logarithm of EC_{50} in mM, and f is a scaling factor, which was set as 20.6 to calibrate the highest (previously known) activator efficiency as 10.[8]

Dynamic light scattering: DLS experiments of the vesicles were carried out on a Malvern Instruments DTS Nano 2000 Zeta-Sizer. A cluster concentration of 150 µM was selected since this concentration lies above the EC_{50} activity for the evaluated clusters as carriers (Table S1). Note that DLS measurements of neat carrier and cargo solutions were also measured; no signal was detected, indicating the absence of any nanoaggregate formation.

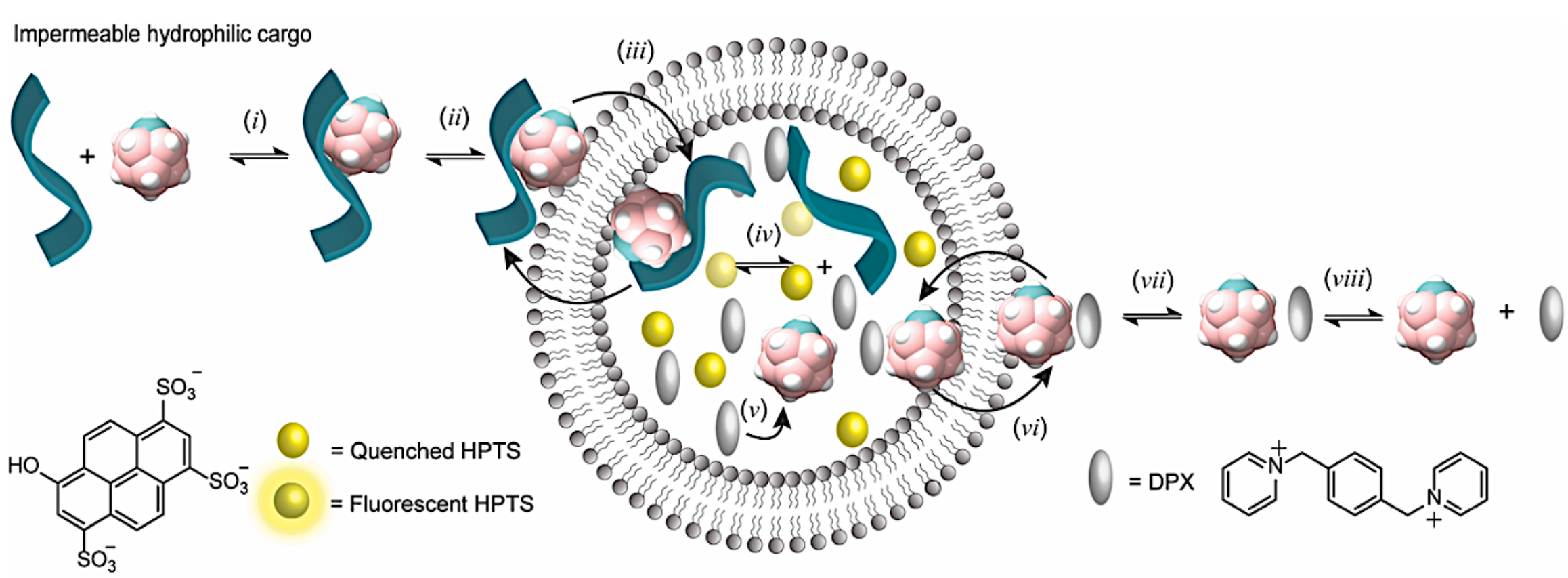

Scheme S1: Schematic representation of the proposed transport mechanism of impermeable hydrophilic cargos carried by the carba-*closo*-dodecaborate cluster **1**, signalled by the HPTS/DPX assay, along with the chemical structures of the fluorescent dye (HPTS) and quencher (DPX). Cluster and cargo form a supramolecular complex (*i*), which interacts with the membrane (*ii*) and crosses through the lipidic bilayer (*iii*). In the vesicular interior, the complex dissociates due to the reversible nature of the binding (*iv*), leaving the cluster free to interact with the positively charged quencher (*v*). Eventually, the complex cluster•quencher complex shuttles back through the membrane (*vi*), resulting in an continuous increase of HPTS fluorescence by effective removal of quencher from the interior of the vesicle.

5. Cellular uptake

CHO-K1 cells were plated into µ-Slide 8-well plates (ibidi) at a density of ≈1 × 10^5 cells per well (per 1 mL) and incubated for 24 h in Ham's F-12 medium containing 10% v/v fetal bovine serum and 1.0% penicillin-streptomycin at 37 °C in a 5% CO_2 atmosphere. The cells were washed twice with Hank's buffer, and incubated for 2 min at 37 °C in Ham's F-12 medium without phenol red with boron cluster carrier (5 µM) and TAMRA-Arg$_8$ (5 µM) for 30 min at 37 °C. Cells incubated without carrier were used as control. The cells were washed twice with Hank's buffer containing 100 µg mL^{-1} heparin and then washed twice with Ham's F-12 medium without phenol red. Nuclear staining was performed by incubating the cells with 10 µg mL^{-1} Hoechst 33 342 (bis-benzimide H33342 trihydrochloride) for 10 min at 37 °C. Cells were imaged by an LSM 980 S6 Airscan2 confocal laser scanning microscope (Zeiss, Germany) and images were processed with the instrument-specific software ZEN blue 3.2.

For endosomal uptake inhibition studies, CHO-K1 cells were seeded in μ-Slide 8-well chamber slides (ibidi) at a density of ≈ 1 × 10^5 cells per well (per 1 mL) in Ham's F-12 medium supplemented with 10% (v/v) fetal bovine serum (FBS) and 1.0% penicillin–streptomycin. Cells were incubated for 24 h at 37 °C in a humidified atmosphere containing 5% CO_2 to allow for proper adhesion. Following incubation, the medium was removed and the cells were washed twice with Hank's balanced salt buffer. Cells were then pre-treated with the respective endocytosis inhibitor, either 40 μM dynasore or 20 μM chlorpromazine, each in phenol-red-free Ham's F-12 medium for 15 min at 37 °C. Subsequently, the cells were incubated with 5 μM of the respective carrier ($CB_{11}H_{12}^{-}$, o-COSAN⁻, $B_{12}H_{12}^{2-}$ and $B_{12}Br_{12}^{2-}$) for 2 min. Thereafter, TAMRA-Arg_8 was added to a final concentration of 5 μM and incubation was continued for 30 min at 37 °C. Cells treated with TAMRA-Arg_8 in the absence of carrier served as controls. Following incubation, the cells were washed twice with Hank's buffer containing 100 μg·mL^{-1} heparin and then twice with Dulbecco's phosphate-buffered saline (PBS) buffer. Nuclear staining was performed by incubating cells with Hoechst 33342 (10 μg mL^{-1}) for 5–10 min at 37 °C in phenol-red-free Ham's F-12 medium, followed by washing twice with PBS buffer. Live-cell imaging was performed at 37 °C in phenol-red-free Ham's F-12 medium immediately afterwards using an LSM 980 S6 confocal laser scanning microscope equipped with an Airyscan2 module (Zeiss, Germany). Images were processed and analyzed with the instrument-specific software ZEN blue 3.2.

For the flow cytometry experiments, CHO-K1 cells were incubated analogously with carrier and TAMRA-Arg_8 (5 μM) for 30 mins. After medium removal and washing, (≈ 1 × 10^5 cells/well per 1 mL) cells were treated with trypsin (300 μL) for 5 min at 37ºC, neutralized with 500 μL Ham´s F-12 medium, washed twice with PBS and resuspended in 1.2 mL PBS. Cells treated only with PBS were used as control. The population of cells with characteristic forward-and side-scatter (FSC/SSC) parameters was selected, and the geometric mean fluorescence intensity (MFI) of the TAMRA channel (λ_{ex} = 552 nm and λ_{em} = 578 nm) was measured on a Sysmex CyFlow Space flow cytometer (Sysmex, Norderstedt, Germany). Data were analyzed with FlowJo software (version 10.10; BD Biosciences).

6. Cellular viability

CHO-K1 cells were cultured in Ham's F-12 medium containing 10% (v/v) fetal bovine serum (FBS) and 1.0% (v/v) penicillin–streptomycin at 37 °C in a humidified atmosphere containing 5% CO_2. Cells were dispersed in a 96-well flat-bottom tissue culture treated plates (Eppendorf) at a density of ca. 5.0 × 10^3 cells per well (per 100 μL) and incubated in Ham's F-12 medium for 12 h at 37 °C, 5% CO_2 to allow cell adhesion.

Cells were subsequently incubated for 24 hours with different concentrations of the boron cluster in Ham´s F-12 medium. After 24 h incubation time, cells were washed twice with PBS and fresh Ham´s F-12 medium (100 μL) was added to each well, then 20 μL of resazurin solution (0.15 mg/mL in PBS) was added and further incubated for 3 h at 37ºC. Fluorescence was measured by using a plate reader with λ_{ex} = 560 nm and λ_{em} = 590 nm on a JASCO FP-8500 spectrofluorometer.

Cell viability was calculated as follows:

$$Cell\ viability = \frac{FI_{\ sample} - FI_{\ blank}}{FI_{\ control} - FI_{\ blank}} \times 100\% \qquad (4)$$

where, FI_{sample} is the fluorescence intensity of cells incubated with the boron clusters, FI_{blank} is the fluorescence intensity of the neat medium, and $FI_{control}$ denotes the fluorescence of untreated cells (without cluster). Each experiment was performed as triplicates.

7. Computational details

The structures of the boron clusters and supramolecular inclusion complexes of cyclodextrins with $CB_{11}H_{12}^{-}$ were obtained from density functional theory (DFT) optimizations. Geometry optimizations and polarizability calculations of the boron clusters were performed at the B3LYP-D3/6-311+G(2d,p) level of theory,[9,10] which has shown good agreement with experimental data in previous studies.[11] Only for the cobalt metal center was the Hay-Wadt Los Alamos relativistic effective core potential with a double-ζ basis set, augmented by d-type polarization and p-type diffuse functions (LANL2DZdp), employed. [12] Dispersion corrections were included in all DFT calculations using Grimme's D3 model.[13]

The structures of the supramolecular inclusion complexes were optimized using the Minnesota hybrid meta-GGA exchange-correlation functional M06-2X-D3, which has demonstrated reliable performance for noncovalent interactions.[14] Pople-type basis sets were used, but in a reduced form (3-21G or 3-21+G) due to the size of the systems studied. The effect of water solvent, consistent with the experimental conditions, was included implicitly using the SMD solvation model.[15] Multiple initial orientations of $CB_{11}H_{12}^{-}$ relative to the cyclodextrins were considered, particularly the orientation of the carbon atom and the placement of the cluster near the upper and lower rim of the cyclodextrin cavity. Stoichiometries were kept consistent with the ITC experiments: a 2:1 macrocycle:cluster ratio for α-CD and a 1:1 ratio for β- and γ-CD.

All optimized structures were confirmed as minima by the absence of imaginary harmonic vibrational frequencies. DFT calculations were carried out using the Gaussian 16 program package (Revision C.01). [16] Molecular volumes and surface areas were calculated as van-der-Waals values by using the QSAR module integrated in the HyperChem program;[17] surface areas were obtained by the "grid" method. Molecular images were generated with PyMOL software.[18]

8. Cloud-point experiments

Cloud-point experiments were performed on a Cary 4000 UV-vis spectrometer equipped with a Peltier controller (with a thermocouple to measure the temperature directly in the sample) in 1-cm path-length quartz cuvettes. The absorbance of a solution of 1% *w*/*w* of hydroxypropylcellulose in water was monitored at 500 nm with rising temperature at a heating rate of 1ºC per minute. The cloud-point is the temperature at which the turbidity becomes noticeable, which is defined by a sharp increase in apparent absorbance, due to light scattering.

9. Results

9.1. NMR

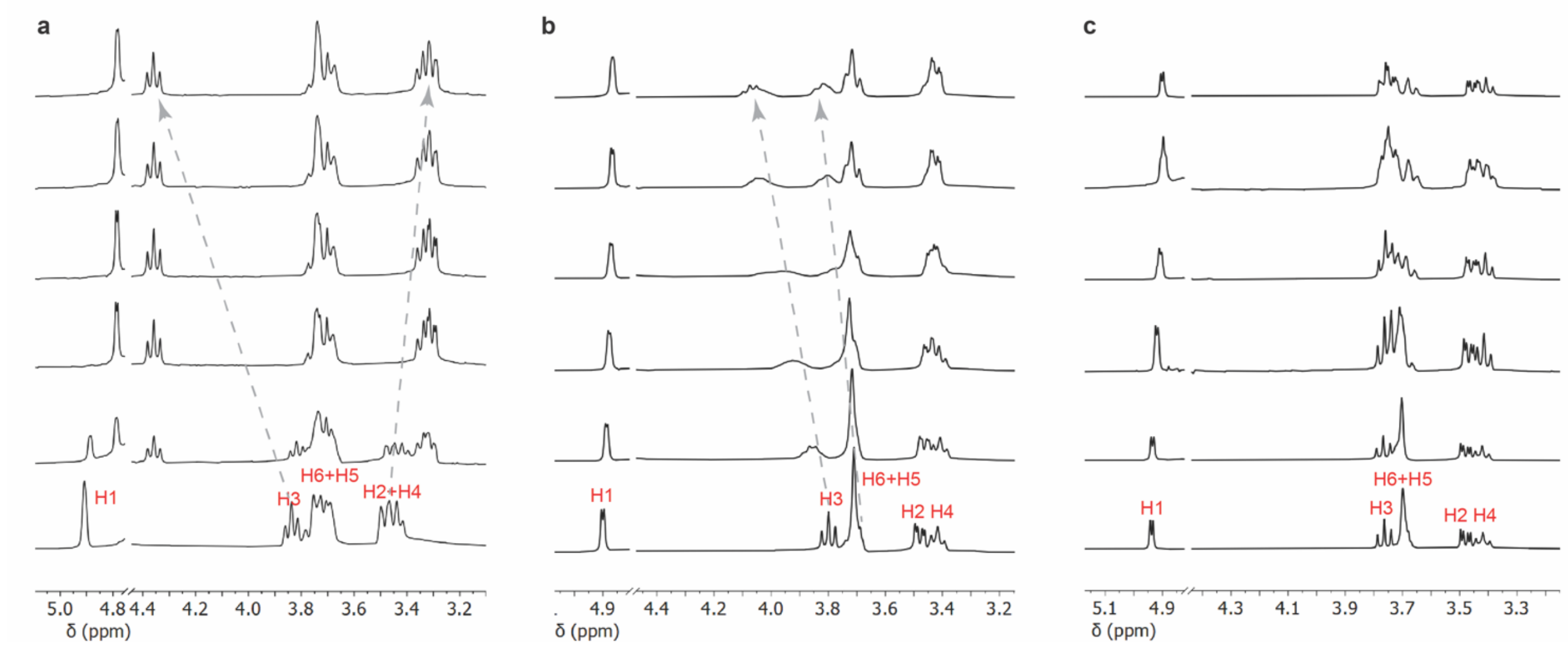

Figure S1: ^{1}H NMR titrations of **a** α-CD (500 μM), **b** β-CD (500 μM), and **c** γ-CD (500 μM), with different concentrations of $CB_{11}H_{12}^{-}$ (**1**, 0-500 μM).

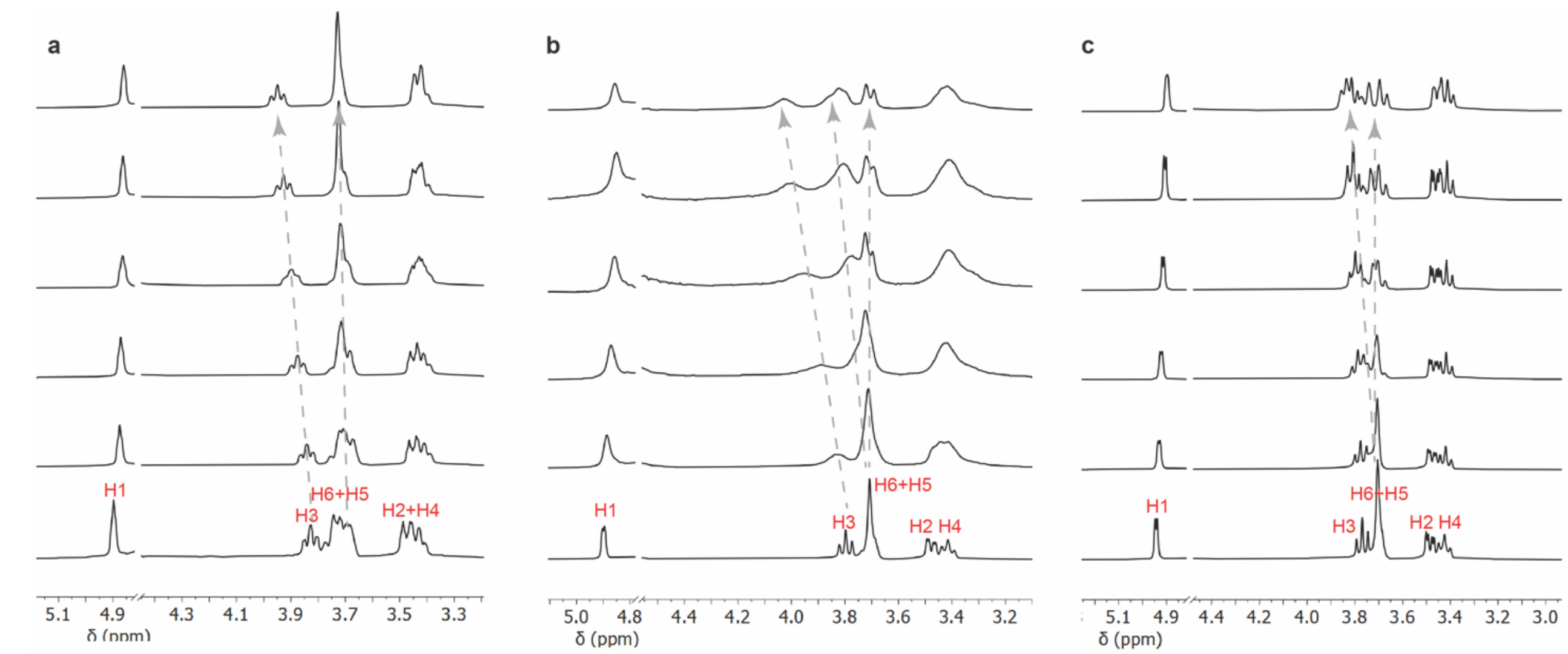

Figure S2: ^{1}H NMR titrations of **a** α-CD (500 μM), **b** β-CD (500 μM), and **c** γ-CD (500 μM), with different concentrations of 12-I-$CB_{11}H_{11}^{-}$ (**2**, 0-500 μM).

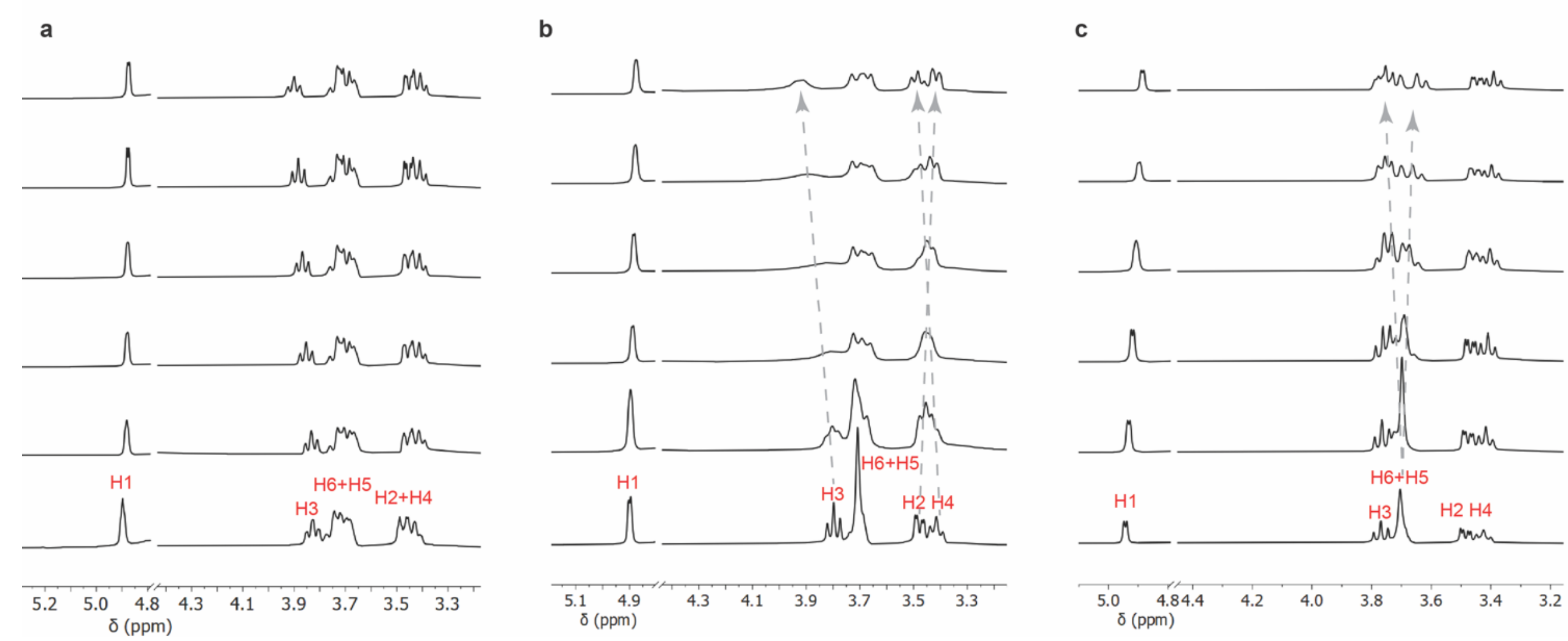

Figure S3: ^{1}H NMR titrations of **a** α-CD (500 μM), **b** β-CD (500 μM), and **c** γ-CD (500 μM), with different concentrations of 12-Ph-$CB_{11}H_{11}^{-}$ (**3**, 0-500 μM).

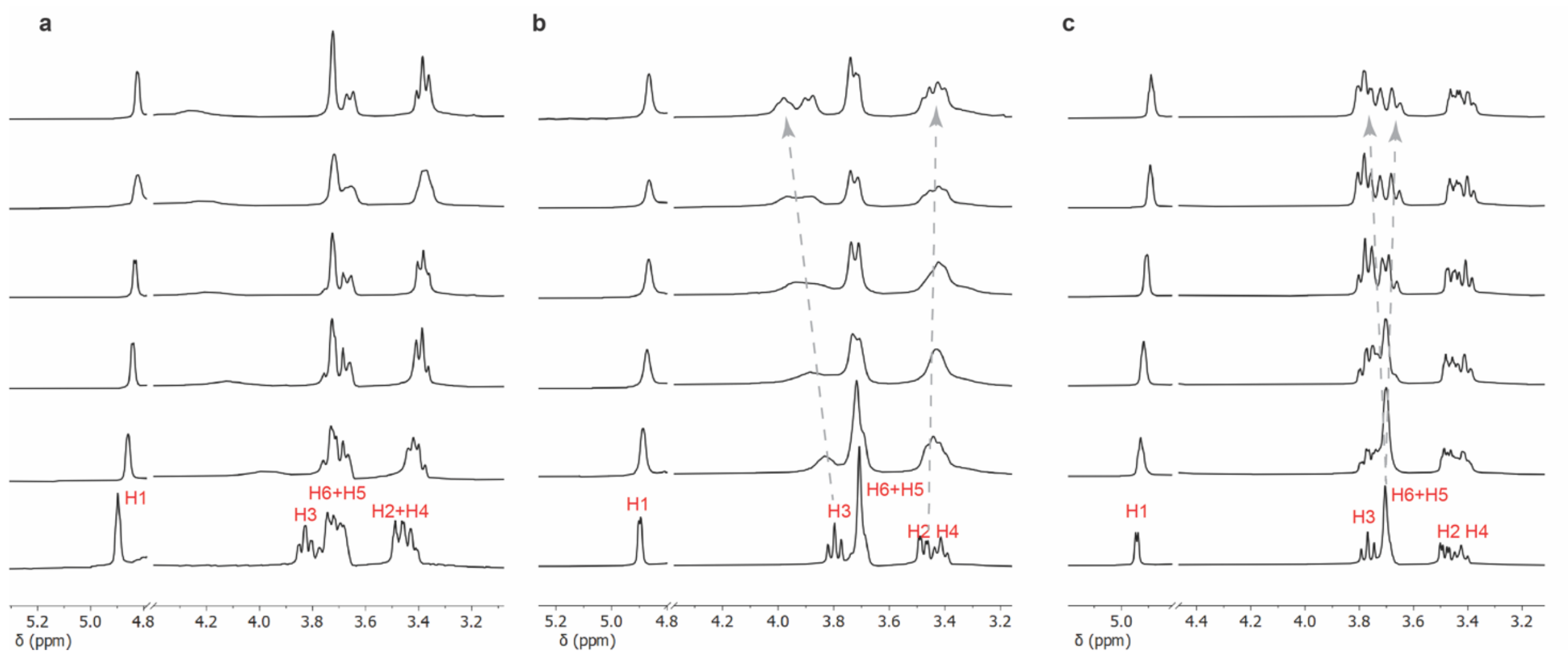

Figure S4: ^{1}H NMR titrations of **a** α-CD (500 μM), **b** β-CD (500 μM), and **c** γ-CD (500 μM), with different concentrations of 12-Et-$CB_{11}H_{11}^{-}$ (**4**, 0-500 μM).

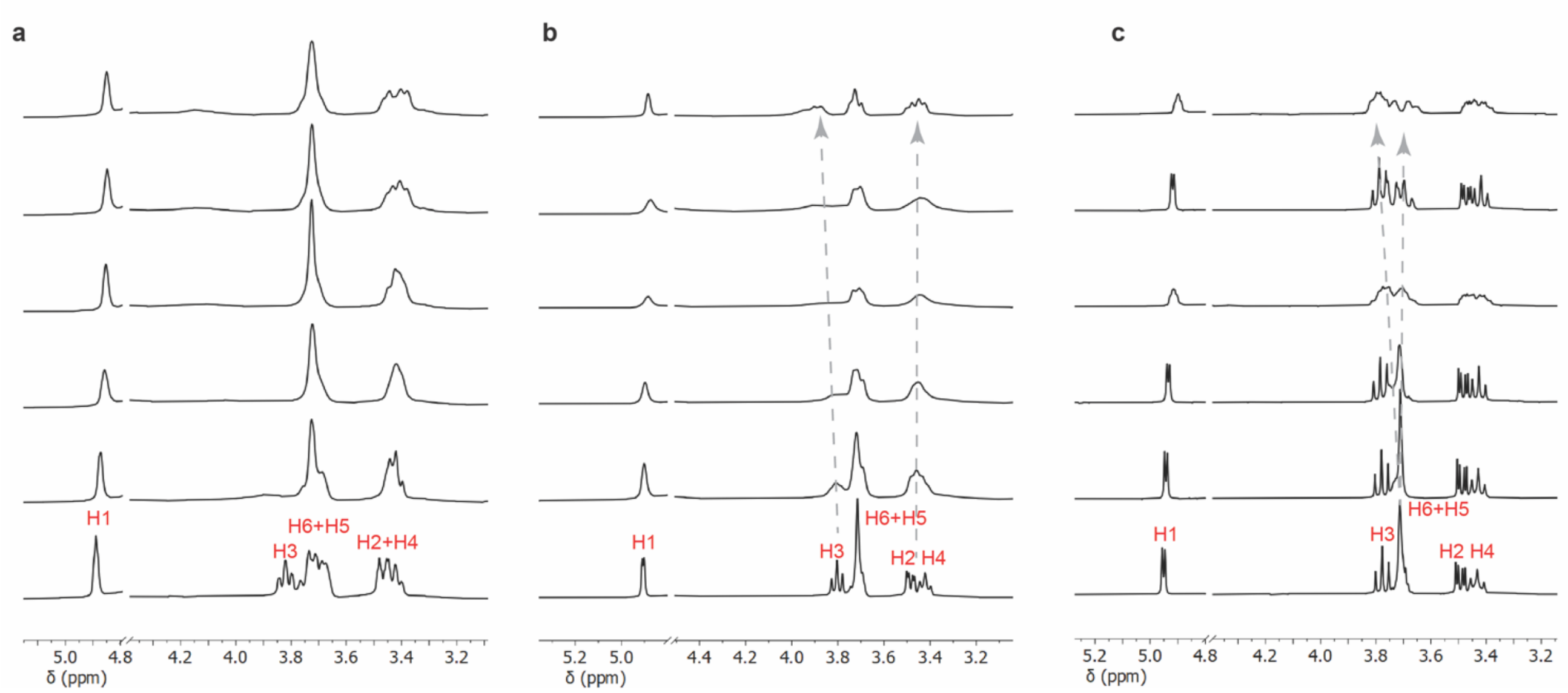

Figure S5: ^{1}H NMR titrations of **a** α-CD (500 μM), **b** β-CD (500 μM), and **c** γ-CD (500 μM), with different concentrations of12-Bu-$CB_{11}H_{11}^{-}$ (**5**, 0-500 μM).

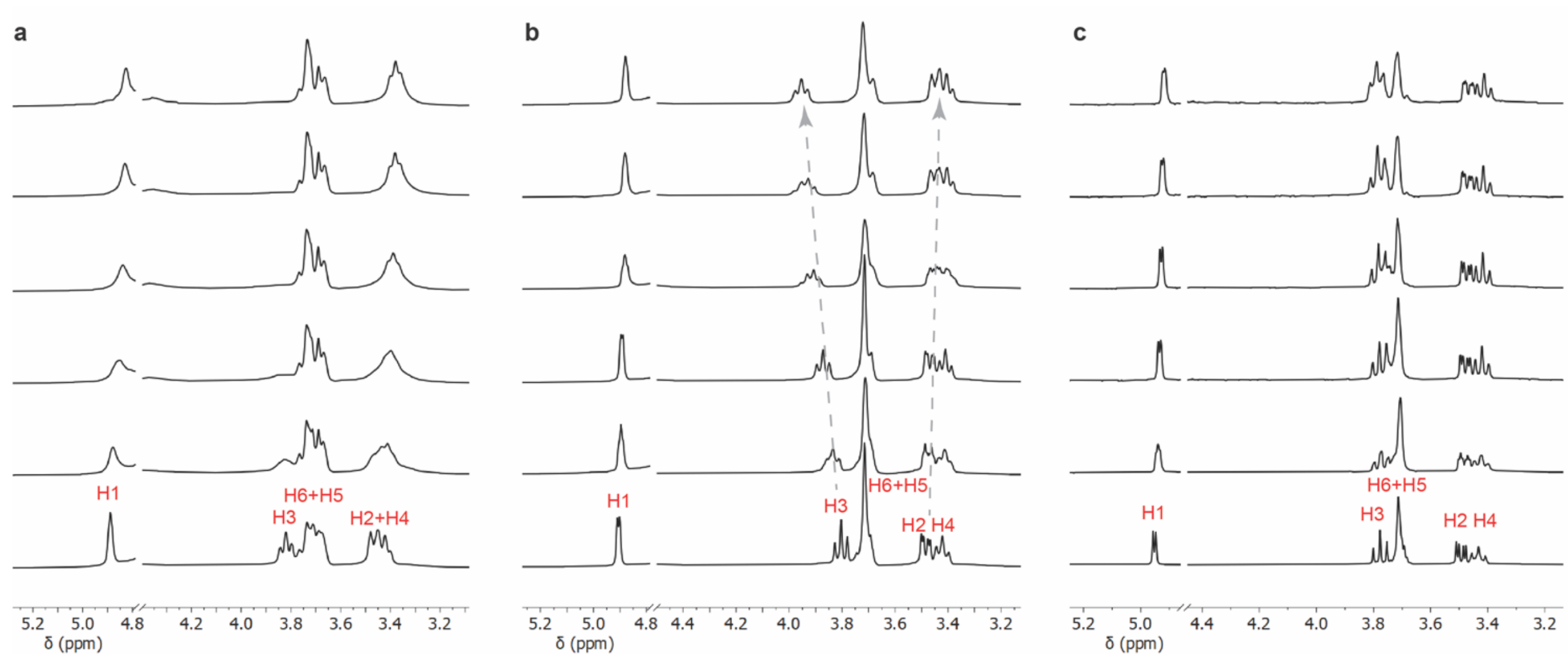

Figure S6: ^{1}H NMR titrations of **a** α-CD (500 μM), **b** β-CD (500 μM), and **c** γ-CD (500 μM), with different concentrations of 12-OH-$CB_{11}H_{11}^{-}$ (**6**, 0-500 μM).

9.2. Molecular Dynamic simulations

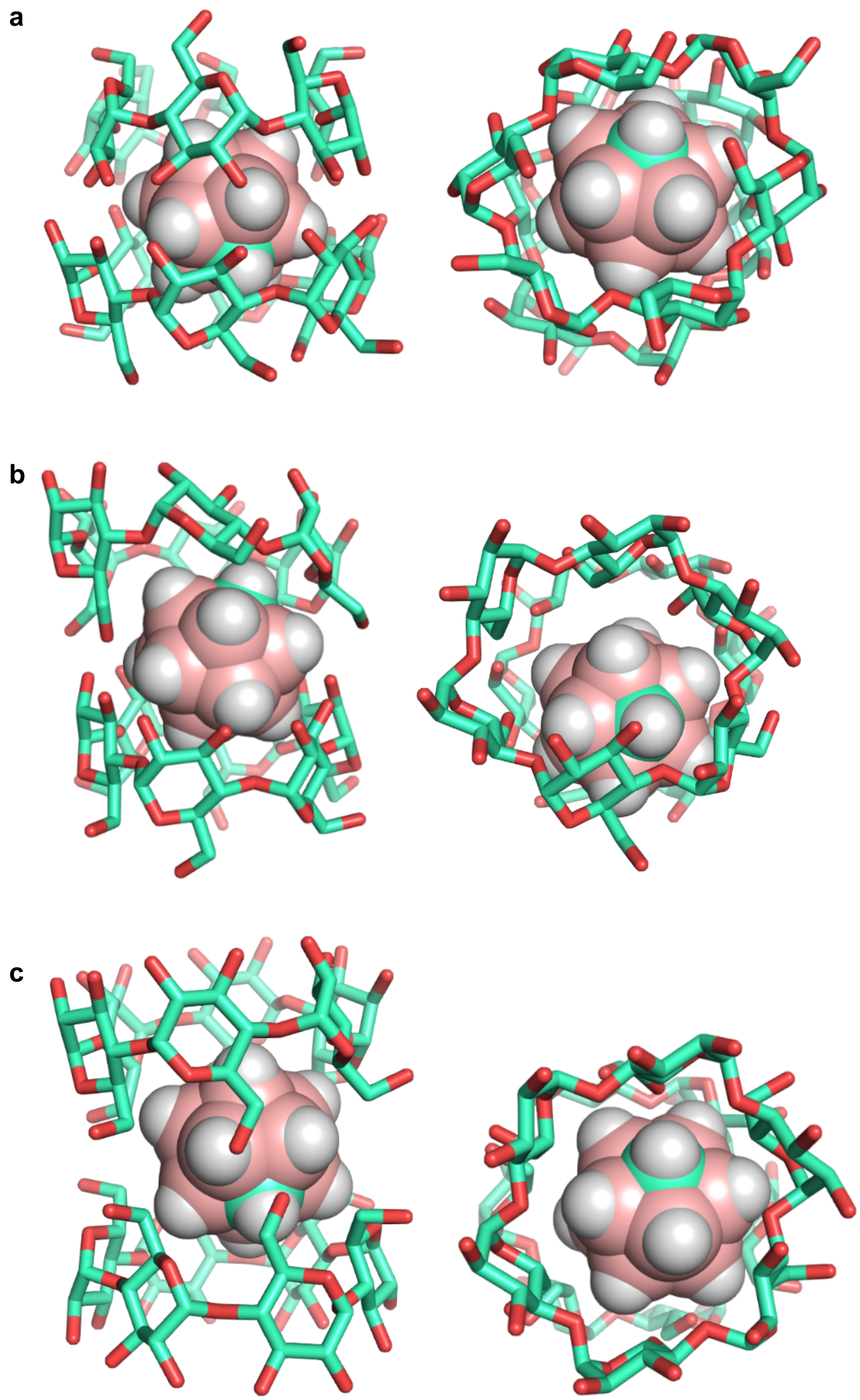

Figure S7. Side and top/bottom views of the geometry-optimized structures (M06-2X-D3/3-21G level of theory, SMD solvation model for water) of ternary 2:1 α-CD•$CB_{11}H_{12}^{-}$ inclusion complexes in water, **a** with the secondary (wider) rims of α-CD forming the capsule (most stable, $\Delta G_{calc,rel} \equiv 0$ kcal mol^{-1}), **b** with one secondary and one primary rim of α-CD forming the capsule (less stable, $\Delta G_{calc,rel}$ = +67 kcal mol^{-1}), and **c** with the primary (narrower) rims of α-CD forming the capsule (least stable, $\Delta G_{calc,rel}$ = +110 kcal mol^{-1}). The ternary capsule **a** is energetically favorable.

Table S1 Thermochemical data of selected ternary 2:1 α-CD•$CB_{11}H_{12}^{-}$ inclusion complexes, see Figure S7, obtained at the M06-2X-D3/3-21G and 3-21+G (in parentheses) level of theory (SMD model for solvation in water) expressed in a.u. ε_0 – electronic energy, ε_{ZPE} – zero-point correction, E_{tot} – thermal correction to energy, H_{corr} – thermal correction to enthalpy, G_{corr} – thermal correction to Gibbs free energy, Δ values – expressed relative to most stable conformation.

	**Complex a**	**Complex b**	**Complex c**
ε_0	-7603.99258317<br>(-7605.54671883)	-7603.88129528<br>(-7605.45034193)	-7603.79426865<br>(-7605.43117666)
**Δε_0 / kcal mol^{-1}**	**0.00**	**69.83**<br>**(60.47)**	**124.44**<br>**(72.50)**
ε_{ZPE}	2.278129<br>(2.267209)	2.280597<br>(2.270635)	2.272894<br>(2.262248)
E_{tot}	2.401821<br>(2.385577)	2.408500<br>(2.394297)	2.406658<br>(2.391229)
H_{corr}	2.402765<br>(2.386521)	2.409445<br>(2.395242)	2.407602<br>(2.392173)
G_{corr}	2.134005<br>(2.127872)	2.129983<br>(2.124360)	2.111227<br>(2.105301)
ε_0 + ε_{ZPE}	-7601.714454<br>(-7603.279510)	-7601.600698<br>(-7603.179707)	-7601.521375<br>(-7603.168929)
ε_0 + E_{tot}	-7601.590762<br>(-7603.161142)	-7601.472795<br>(-7603.056045)	-7601.387611<br>(-7603.039948)
ε_0 + H_{corr}	-7601.589818<br>(-7603.160197)	-7601.471851<br>(-7603.055100)	-7601.386667<br>(-7603.039003)
ε_0 + G_{corr}	-7601.858579<br>(-7603.418847)	-7601.751312<br>(-7603.325982)	-7601.683042<br>(-7603.325876)
**$S = (H - G) / T$ (kcal mol^{-1})**	0.57<br>(0.54)	0.59<br>(0.57)	0.62<br>(0.60)
**ΔG / kcal mol^{-1}**	**0.00**	**67.31**<br>**(58.27)**	**110.15**<br>**(58.34)**

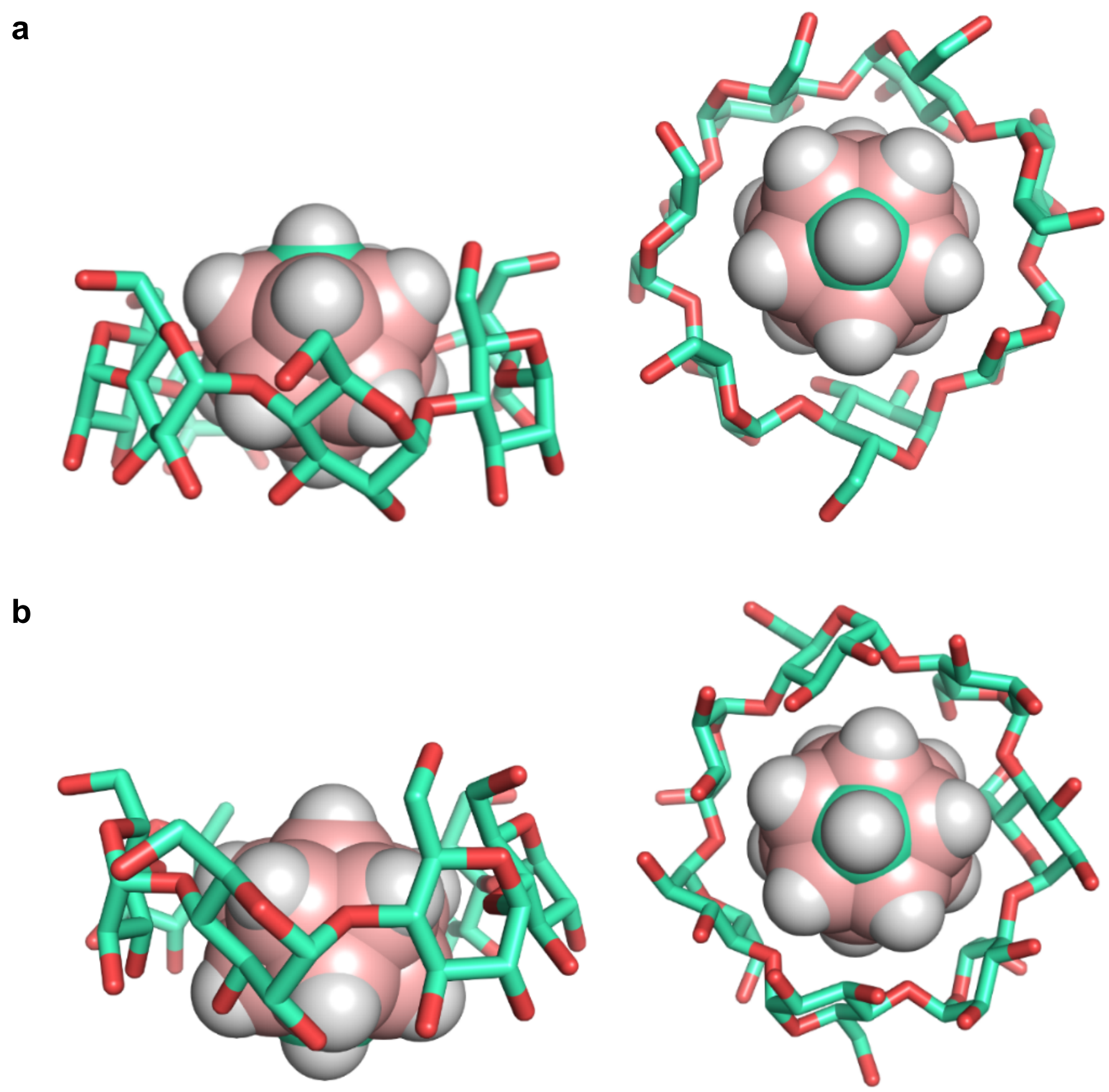

Figure S8. Side and top/bottom views of the geometry-optimized structures (M06-2X-D3/3-21G level of theory, SMD solvation model for water) of binary 1:1 β-CD•$CB_{11}H_{12}^{-}$ inclusion complexes in water, **a** with the C–H bond of $CB_{11}H_{12}^{-}$ protruding towards the primary (narrower) rim of β-CD forming the capsule (most stable, $\Delta G_{calc,rel} \equiv 0$ kcal mol^{-1}) and **b** with the C–H bond of $CB_{11}H_{12}^{-}$ protruding towards the secondary (wider) rim of β-CD forming the capsule (less stable, $\Delta G_{calc,rel}$ = +6.6 kcal mol^{-1}). Complex **a** is energetically more favorable.

Table S2. Thermochemical data of selected binary 1:1 β-CD•$CB_{11}H_{12}^{-}$ inclusion complexes, see Figure S8, obtained at the M06-2X-D3/3-21G and 3-21+G (in parentheses) level of theory (SMD model for solvation in water) expressed in a.u. ε_0 – electronic energy, ε_{ZPE} – zero-point correction, E_{tot} – thermal correction to energy, H_{corr} – thermal correction to enthalpy, G_{corr} – thermal correction to Gibbs free energy, Δ values – expressed relative to most stable conformation.

	**Complex a**	**Complex b**
ε_0	-4567.70884003<br>(-4568.66390569)	-4567.70014942<br>(-4568.65647919)
**Δε_0 / kcal mol^{-1}**	**0.00**	**5.45**<br>**(4.66)**
ε_{ZPE}	1.401458<br>(1.396214)	1.402963<br>(1.399050)
E_{tot}	1.483068<br>(1.473287)	1.484212<br>(1.476030)
H_{corr}	1.484012<br>(1.474232)	1.485156<br>(1.476974)
G_{corr}	1.291271<br>(1.293753)	1.293046<br>(1.295597)
$\varepsilon_0 + \varepsilon_{ZPE}$	-4566.307382<br>(-4567.267692)	-4566.297187<br>(-4567.257429)
$\varepsilon_0 + E_{tot}$	-4566.225772<br>(-4567.190618)	-4566.215938<br>(-4567.180449)
$\varepsilon_0 + H_{corr}$	-4566.224828<br>(-4567.189674)	-4566.214993<br>(-4567.179505)
$\varepsilon_0 + G_{corr}$	-4566.417569<br>(-4567.370153)	-4566.407104<br>(-4567.360883)
**$S = (H - G) / T$ (kcal mol^{-1})**	0.41<br>(0.38)	0.40<br>(0.38)
**ΔG / kcal mol^{-1}**	**0.00**	**6.57**<br>**(5.82)**

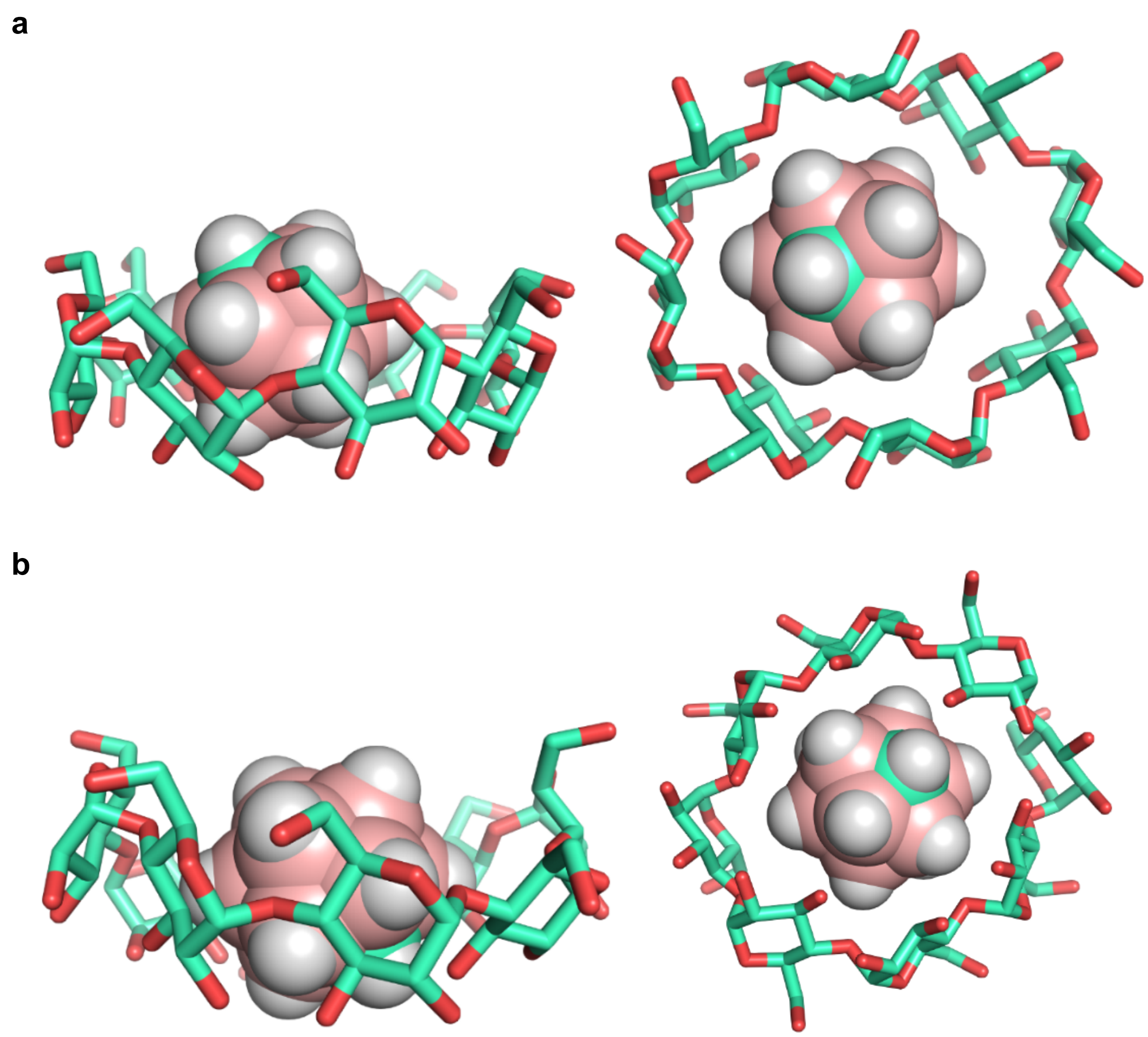

Figure S9. Side and top/bottom views of the geometry-optimized structures (M06-2X-D3/3-21G level of theory, SMD solvation model for water) of binary 1:1 γ-CD•$CB_{11}H_{12}^{-}$ inclusion complexes in water, **a** with the C–H bond of $CB_{11}H_{12}^{-}$ protruding (and tilting) upwards the primary (narrower) rim of γ -CD forming the capsule (most stable, $\Delta G_{\mathrm{calc,rel}} \equiv 0$ kcal mol^{-1}) and **b** with the C–H bond of $CB_{11}H_{12}^{-}$ protruding (and tilting) downwards towards the secondary (wider) rim of γ -CD forming the capsule (less stable, $\Delta G_{\mathrm{calc,rel}}$ = +3.4 kcal mol^{-1}). Complex **a** is energetically more favorable.

Table S3. Thermochemical data of selected binary 1:1 γ-CD•$CB_{11}H_{12}^{-}$ inclusion complexes, see Figure S9, obtained at the M06-2X-D3/3-21G and 3-21+G (in parentheses) level of theory (SMD model for solvation in water) expressed in a.u. ε_0 – electronic energy, $\varepsilon_{\mathrm{ZPE}}$ – zero-point correction, E_{tot} – thermal correction to energy, H_{corr} – thermal correction to enthalpy, G_{corr} – thermal correction to Gibbs free energy, Δ values – expressed relative to most stable conformation.

	**Complex a**	**Complex b**
ε_0	-5174.90386726<br>(-5175.98204833)	-5174.89933339<br>(-5175.96451323)
**$\Delta\varepsilon_0$ / kcal mol^{-1}**	**0.00**	**2.84**<br>**(11.00)**
$\varepsilon_{\mathrm{ZPE}}$	1.579184<br>(1.575398)	1.578597<br>(1.573409)
E_{tot}	1.670775<br>(1.660626)	1.670504<br>(1.661999)
H_{corr}	1.671719<br>(1.661571)	1.671448<br>(1.662943)
G_{corr}	1.456482<br>(1.464493)	1.457510<br>(1.456202)
$\varepsilon_0 + \varepsilon_{\mathrm{ZPE}}$	-5173.324683<br>(-5174.406650)	-5173.320737<br>(-5174.391104)
$\varepsilon_0 + E_{\mathrm{tot}}$	-5173.233093<br>(-5174.321422)	-5173.228829<br>(-5174.302514)
$\varepsilon_0 + H_{\mathrm{corr}}$	-5173.232148<br>(-5174.320478)	-5173.227885<br>(-5174.301570)
$\varepsilon_0 + G_{\mathrm{corr}}$	-5173.447386<br>(-5174.517556)	-5173.441824<br>(-5174.508311)
**$S = (H - G) / T$ (kcal mol^{-1})**	0.45<br>(0.41)	0.45<br>(0.44)
**ΔG / kcal mol^{-1}**	**0.00**	**3.40**<br>**(5.80)**

9.3. Isothermal titration calorimetry (ITC)

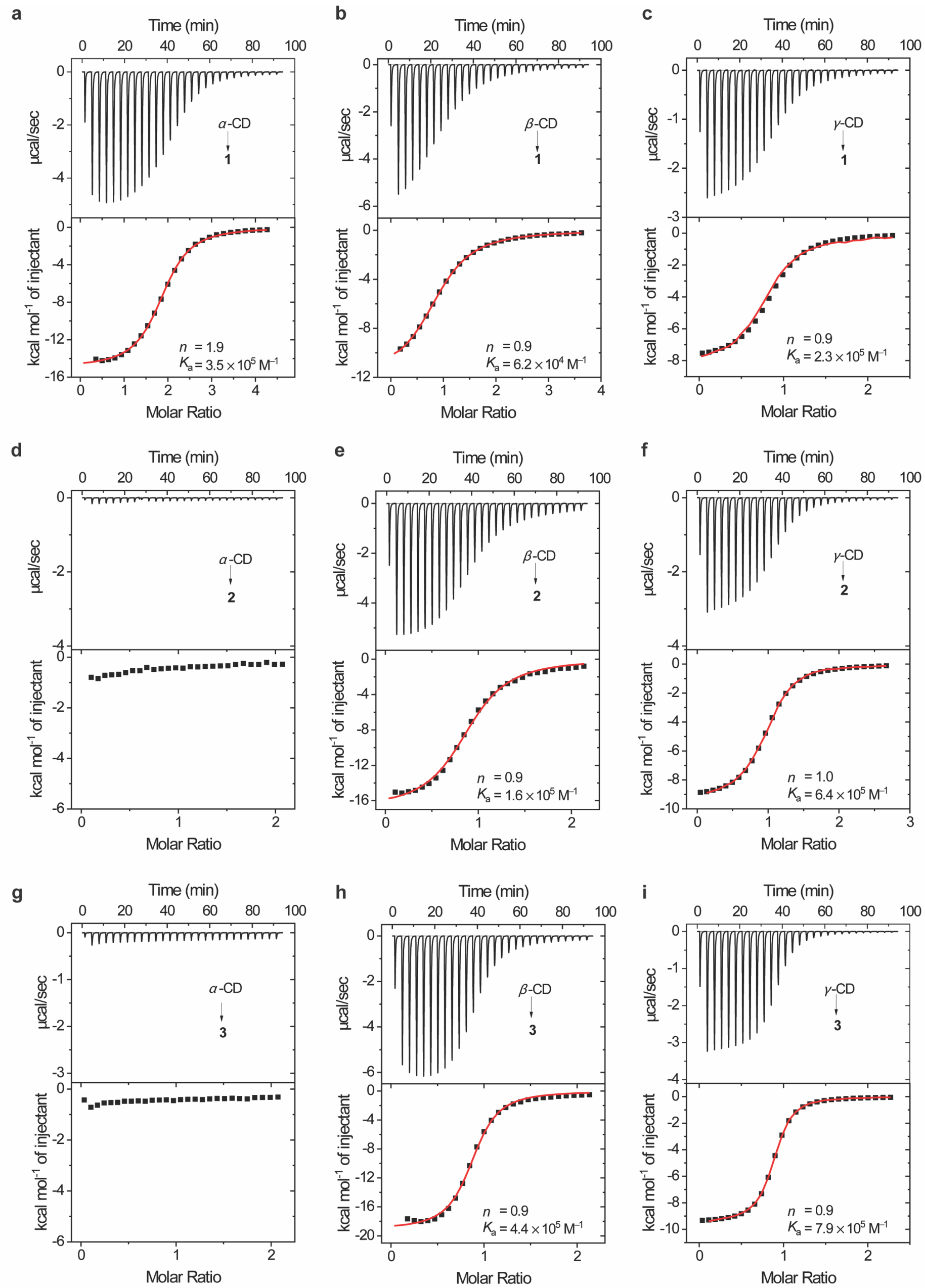

Figure S10: Microcalorimetric titrations of the parent carba-*closo*-dodecaborate (**1**), and the substituted derivatives, 12-I-$CB_{11}H_{12}^-$ (**2**), 12-Ph-$CB_{11}H_{12}^-$ (**3**), and 12-Et-$CB_{11}H_{12}^-$ (**4**), with α-β- and γ-Cyclodextrin (from left to right). Raw ITC data (top) for the sequential injections of cyclodextrin solution into the cluster solution and apparent reaction heats (bottom) obtained from the integration of the calorimetric traces are shown. Cyclodextrin/cluster concentrations in mM: **a**, 1/0.05; **b,** 1.7/0.1; **c**, 1/0.1; **d**, 0.5/0.05; **e**, 1/0.1; **f**, 0.5/0.04; **g**, 1/0.1; **h**, 1/0.1; **i**, 1/0.95; **j**, 1/0.1; **k**, 0.75/0.1; **l**, 0.75/0.1. Error data is 10% for K_a and *n* values (duplicate measurements).

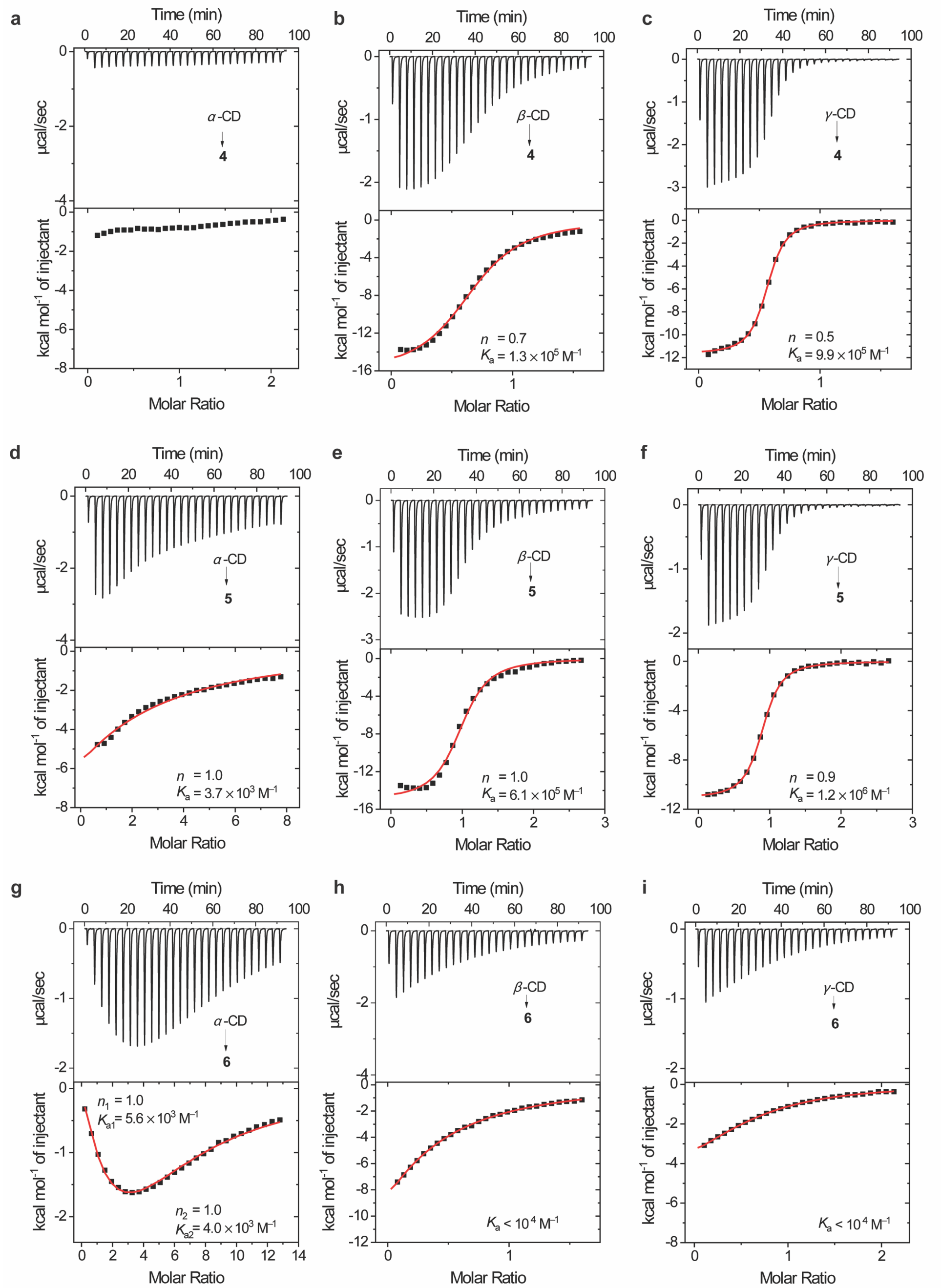

Figure S11: Microcalorimetric titrations of the substituted derivatives, 12-Bu-$CB_{11}H_{12}^-$ (**5**), 12-OH-$CB_{11}H_{12}^-$ (**6**), and 7,12-$(OH)_2$-$CB_{11}H_{12}^-$ (**7**), with α-β- and γ-Cyclodextrin (from left to right). Raw ITC data (top) for the sequential injections of cyclodextrin solution into the cluster solution and apparent reaction heats (bottom) obtained from the integration of the calorimetric traces are shown. Cyclodextrin/cluster concentrations in μM: **a**, 1/0.1; **b,** 0.5/0.04; **c**, 0.5/0.05; **d**, 3/0.05; **e**, 0.75/0.1; **f**, 1/0.1; **g**, 1/0.1; **h**, 1/0.1; **i**, 1/0.1. Error data is 10% for K_a and n values (duplicate measurements).

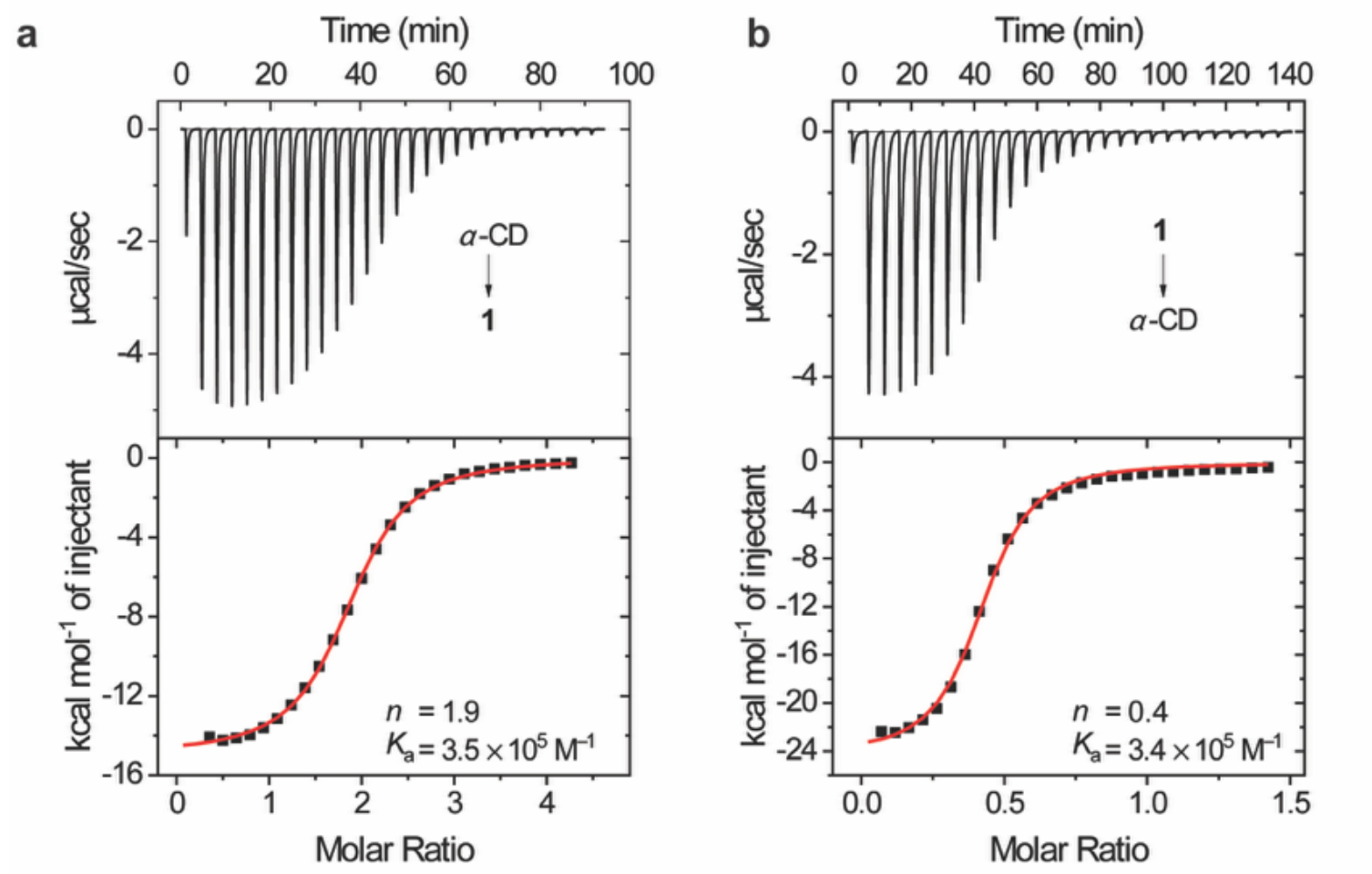

Figure S12: "Direct" (host to guest, left) and reverse (guest to host, right) microcalorimetric titrations of the parent carba-*closo*-dodecaborate (**1**) with α-cyclodextrin. Raw ITC data (top) for the sequential injections of cluster/CD solution into a CD/cluster solution and apparent reaction heats (bottom) obtained from the integration of the calorimetric traces are shown. Syringe/cell concentrations in mM: **a**, 1/0.05; **b,** 1/0.15. Error data is 10% for K_a and *n* values (duplicate measurements).

Table S4. Binding constants (K_a) and thermodynamic data (ΔH and $T\Delta S$) measured for the complex formation between α-CD and the carba-*closo*-dodecaborate anion (**1**) in water.

Titration	*n*	K_a (10^3 M^{-1})	ΔH^o (kcal mol^{-1})	$T\Delta S^o$ (kcal mol^{-1})
direct	1.9	350	−14.9	−7.4
reverse	0.4	342	−24.4	−16.8

Direct titration: α-CD in syringe added to **1** in cell. Reverse titration: **1** in syringe added to α-CD in cell, see Figure S9. Error data is 10% for K_a and *n* values and ± 0.5 kcal mol^{-1} for ΔH and $T\Delta S$ (duplicate measurements).

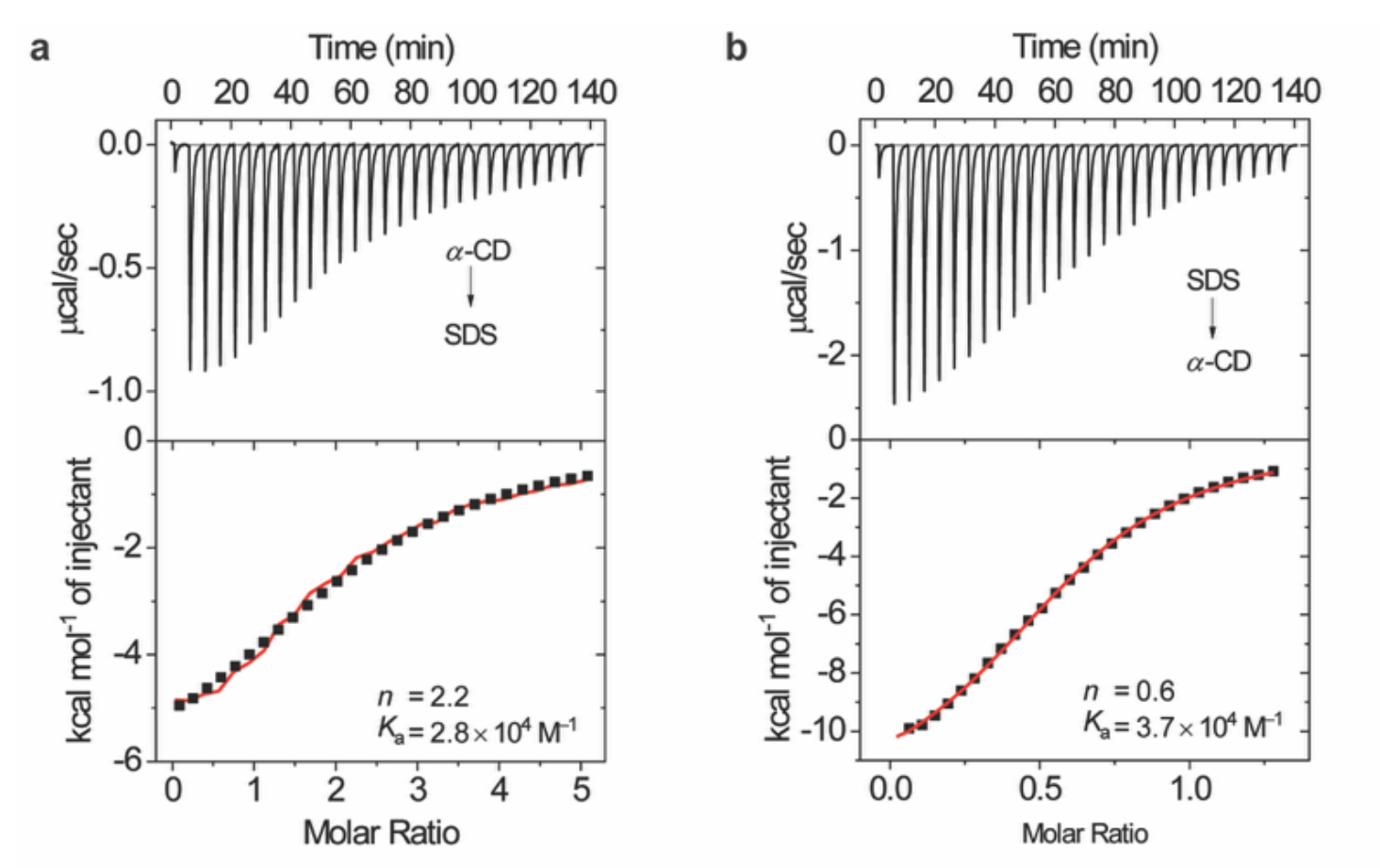

Figure S13: Microcalorimetric titrations of SDS, with α-cyclodextrin. Raw ITC data (top) for the sequential injections of α-CD into SDS solution (**a**) or SDS into a α-CD solution (**b**), and apparent reaction heats (bottom) obtained from the integration of the calorimetric traces. Syringe/cell concentrations in mM: **a**, 1/0.04; **b,** 1/0.15. Error data is 10% for K_a and *n* values.

Table S5. Binding constants (K_a) and thermodynamic data (ΔH and $T\Delta S$) measured for the complex formation between α-CD and SDS in water.

Titration	*n*	K_a (10^3 M^{-1})	ΔH^o (kcal mol^{-1})	$T\Delta S^o$ (kcal mol^{-1})
direct	2.2	28.0	−6.9	−0.8
reverse	0.6	36.6	−12.6	−6.4

Direct titration: α-CD in syringe added to SDS in cell. Reverse titration: SDS in syringe added to α-CD in cell, see Figure S10. Error data is 10% for K_a and *n* values and ± 0.5 kcal mol^{-1} for ΔH and $T\Delta S$ (duplicate measurements).

9.4. Transport experiments in vesicles

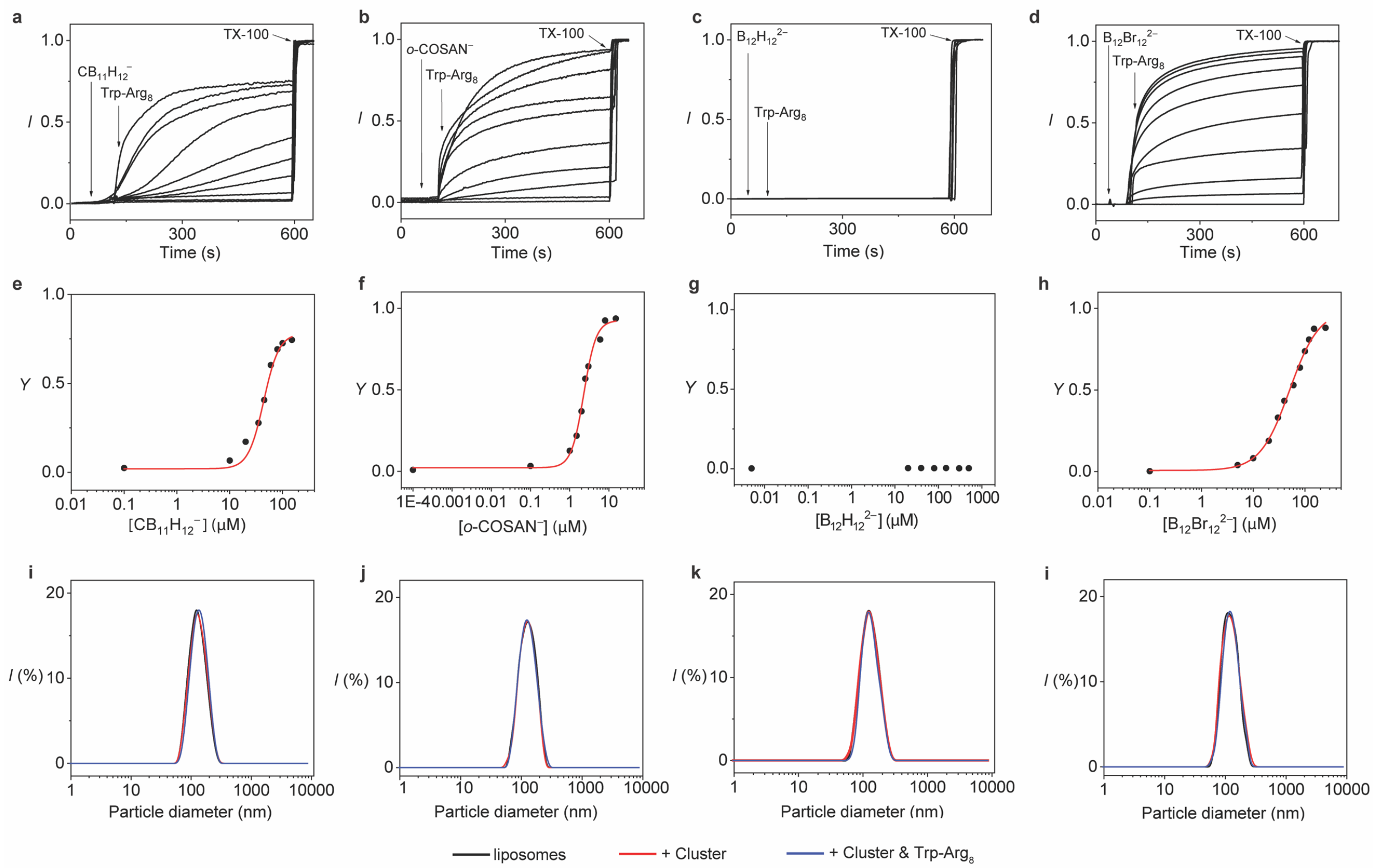

Figure S14: Changes in the fractional dye emission intensity in EYPC liposomes (13 μM phospholipid concentration) as a function of time during the addition of cluster (as carrier, at t = 50 s), octaarginine (as cargo, fixed concentration of 20 μM, at t = 100 s), and TX-100 (for calibration, at t = 600 s, **a**-**d**). Dependence of the fractional activity, Y, *vs* carrier concentration and the corresponding Hill curve fits (**e-h**). Size distribution (by DLS) of liposomes before (black trace), after the addition of cluster alone (red trace), and in presence of octaarginine (blue trace, 20 μM, **i-j**). Experiments with $CB_{11}H_{12}^{-}$, $B_{12}H_{12}^{2-}$, and $B_{12}Br_{12}^{2-}$ were conducted in HPTS/DPX vesicles (λ_{ex} = 413 nm, λ_{em} = 511 nm); for *o*-COSAN⁻ transport experiments CF vesicles (λ_{ex} = 492 nm, λ_{em} = 517 nm) were used.

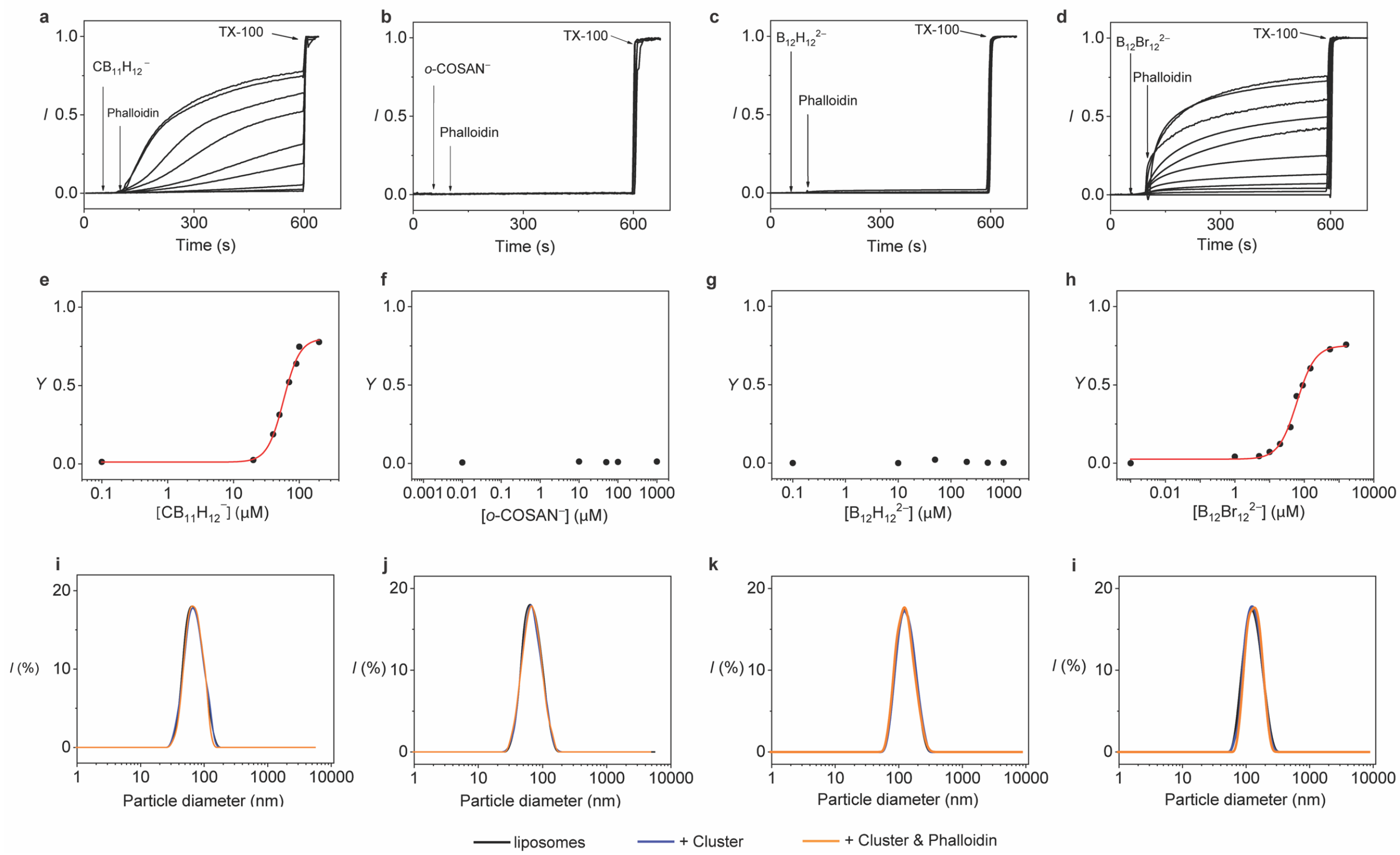

Figure S15: Changes in the fractional dye emission intensity in EYPC liposomes (13 μM phospholipid concentration) as a function of time during the addition of cluster (as carrier, at t = 50 s), octaarginine (as cargo, fixed concentration of 20 μM, at t = 100 s), and TX-100 (for calibration, at t = 600 s, **a**-**d**). Dependence of the fractional activity, Y, *vs* carrier concentration and the corresponding Hill curve fits (**e-h**). Size distribution (by DLS) of liposomes before (black trace), after the addition of cluster alone (blue trace), and in presence of phalloidin (orange trace, 20 μM, **i-j**). Experiments with $CB_{11}H_{12}^-$, $B_{12}H_{12}^{2-}$, and $B_{12}Br_{12}^{2-}$ were conducted in HPTS/DPX vesicles (λ_{ex} = 413 nm, λ_{em} = 511 nm); for *o*-COSAN⁻ transport experiments CF vesicles (λ_{ex} = 492 nm, λ_{em} = 517 nm) were used.

Table S6: Characteristic membrane transport parameters of boron clusters for selected impermeable peptide cargos[a]

Boron cluster carrier	Octaarginine				Phalloidin			
	Y_{max} (%)	EC_{50} (μM)	n	E	Y_{max} (%)	EC_{50} (μM)	n	E
$CB_{11}H_{12}^-$ [b]	78.0	43.9	2.9	5.1	80.1	57.6	3.5	4.8
o-COSAN⁻ [c]	92.6	2.3	2.7	11.9	---[d]	---[d]	---[d]	---[d]
$B_{12}H_{12}^{2-}$ [c]	---[d]	---[d]	---[d]	---[d]	---[d]	---[d]	---[d]	---[d]
$B_{12}Br_{12}^{2-}$ [c]	99.1	50.4	1.5	6.2	75.1	60.8	1.6	4.3

[a] All experiments were conducted with 20 μM cargo. [b] Used as trimethylammonium salt. [c] Used as sodium salt. [d] no detectable activity. 5% (SD) error for Y_{max}, EC_{50}, and n. 10% error for E_a (SD, calculated by considering error propagation with respect to both, Y_{max} and EC_{50}).

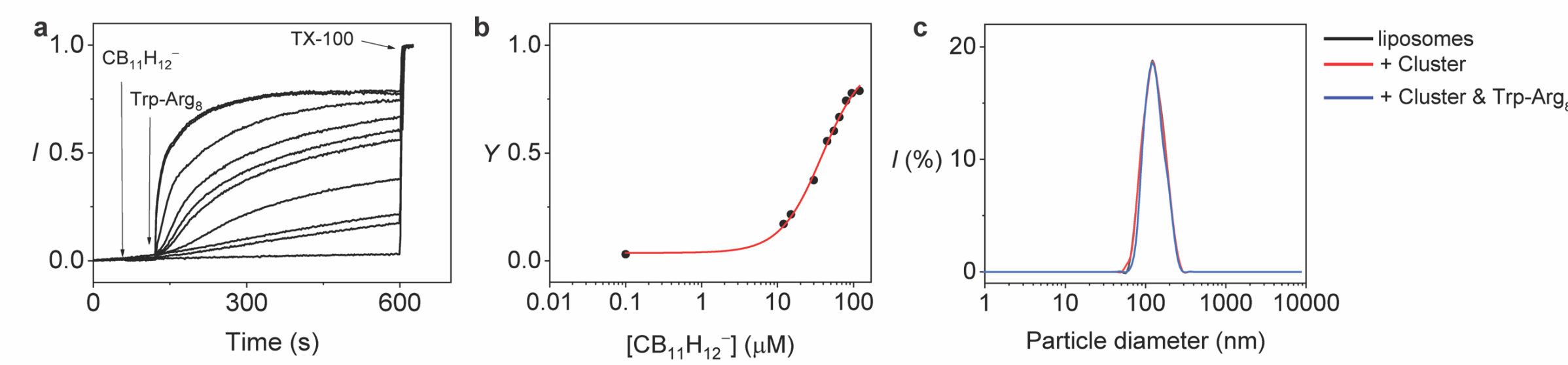

Figure S16: Control experiment with an independent sample of $NaCB_{11}H_{12}$ (commercial, sodium instead of trimethylammonium salt)**.** **a** Changes in the fractional dye emission intensity in EYPC liposomes (13 μM phospholipid concentration, λ_{ex} = 413 nm, λ_{em} = 511 nm) as a function of time during the addition of different concentrations of $NaCB_{11}H_{12}$ (as carrier, at *t* = 50 s), Trp-Arg_6 (as cargo, fixed concentration of 20 μM, at *t* = 100 s), and TX-100 (for calibration, at *t* = 600 s). **b** Dependence of the fractional activity, *Y*, *vs* cluster concentration and the corresponding Hill curve fit. **c** Size distribution (by DLS) of liposomes before (black trace), after the addition of $NaCB_{11}H_{12}$ alone (red trace), and in presence of octaarginine (blue trace, 20 μM).

Table S7: Characteristic transmembrane transport parameters[a] for different salts of $CB_{11}H_{12}^-$ (different counter cations) as molecular carriers of octaarginine (20 μM), determined by HPTS/DPX assay in EYPC vesicles.

Counter cation	Y_{max} (%)	EC_{50} (μM)	*n*	*E*
$N(CH_3)_3H^+$	78.0	43.9	2.9	5.1
Na^+	80.4	39.4	2.6	5.4

[a] 5% (SD) error for Y_{max}, EC_{50}, and *n*. 10% error for E_a (SD, calculated by considering error propagation with respect to both, Y_{max} and EC_{50}).

9.5. Cellular uptake

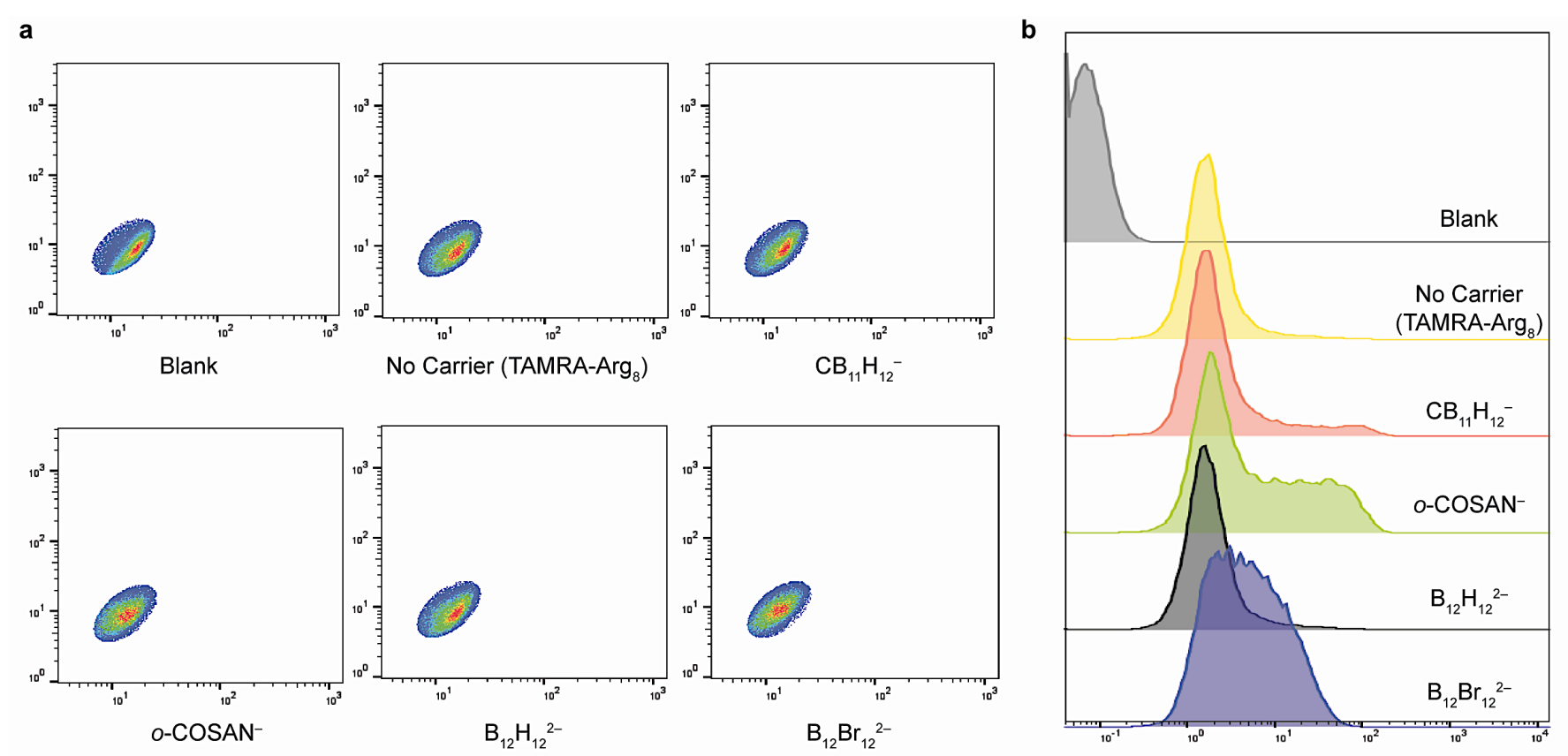

Figure S17: **a** Gating strategy for one of the three replicates (axes: x = FSC, y = SSC). **b** Normalized histograms showing TAMRA fluorescence in the gated population for one of the replicates. Concentrations were 5 μM TAMRA-Arg_8 and 5 μM cluster.

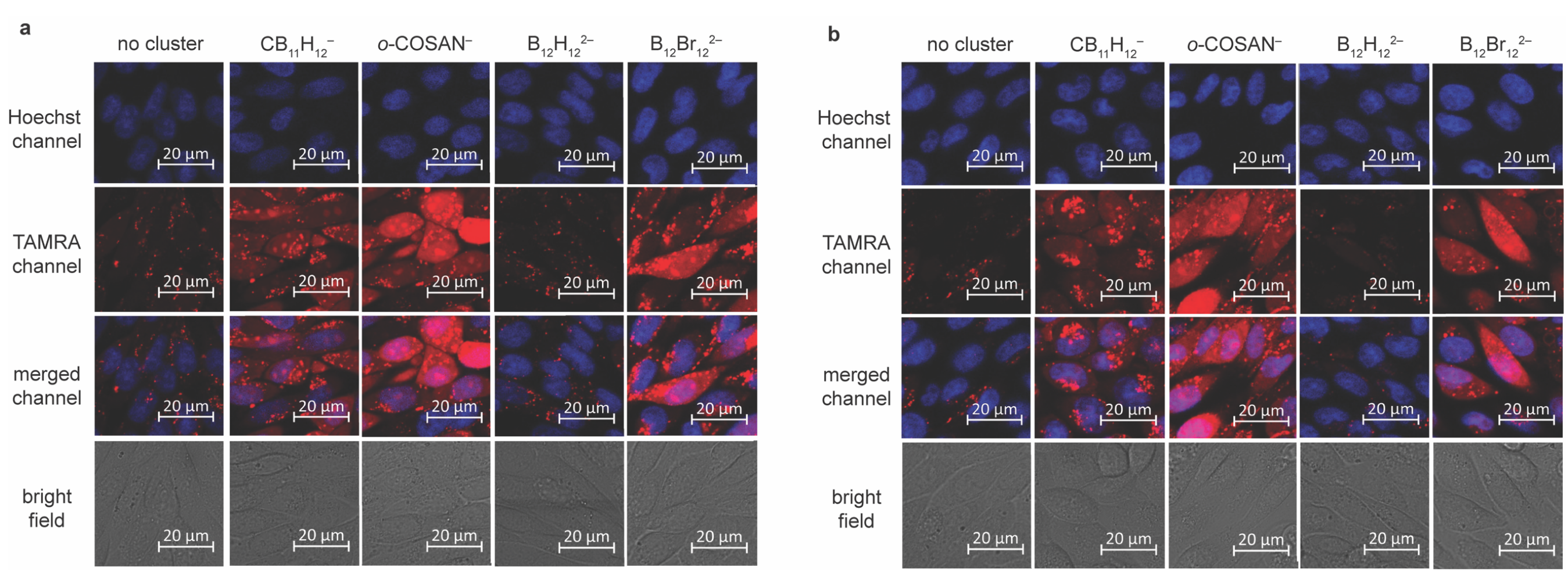

Figure S18: Confocal images of live CHO-K1 cells pre-treated with **a** 40 μM dynasore, or **b** 20 μM chlorpromazine for 15 minutes, followed by incubation with 5 μM TAMRA-Arg_8 alone (no carrier) and in the presence of boron clusters: $CB_{11}H_{12}^-$, *o*-COSAN−, $B_{12}H_{12}^{2-}$, and $B_{12}Br_{12}^{2-}$ (all 5 μM).

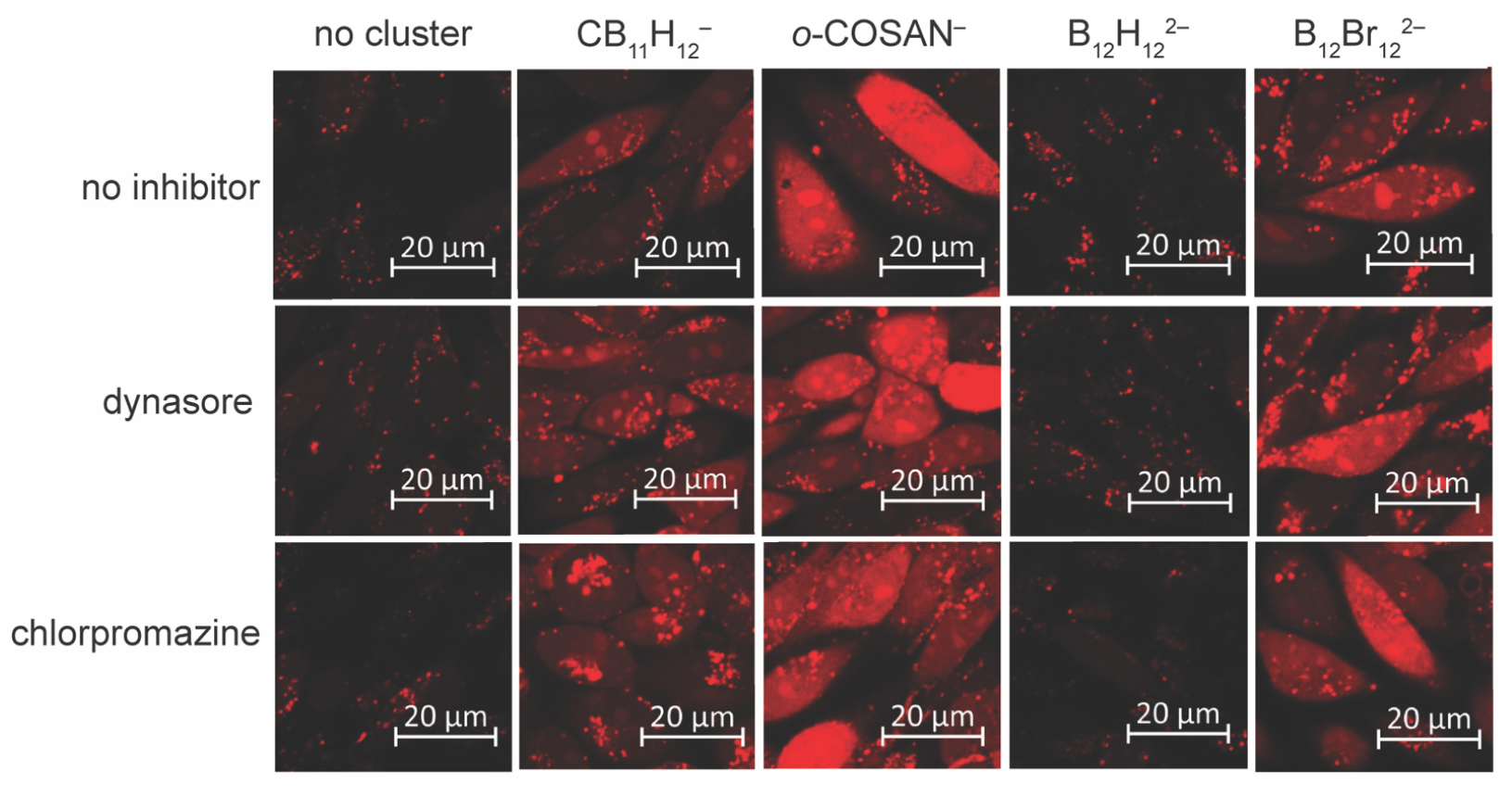

Figure S19: Confocal images,of live CHO-K1 cells pre-treated with different endocytosis inhibitors (40 µM dynasore, or 20 µM chlorpromazine) for 15 minutes, followed by incubation with 5 µM TAMRA-Arg$_8$ alone (no carrier) and in the presence of boron clusters: $CB_{11}H_{12}^{-}$, *o*-COSAN^{-}, $B_{12}H_{12}^{2-}$, and $B_{12}Br_{12}^{2-}$ (all 5 µM).

9.6. Cellular viability

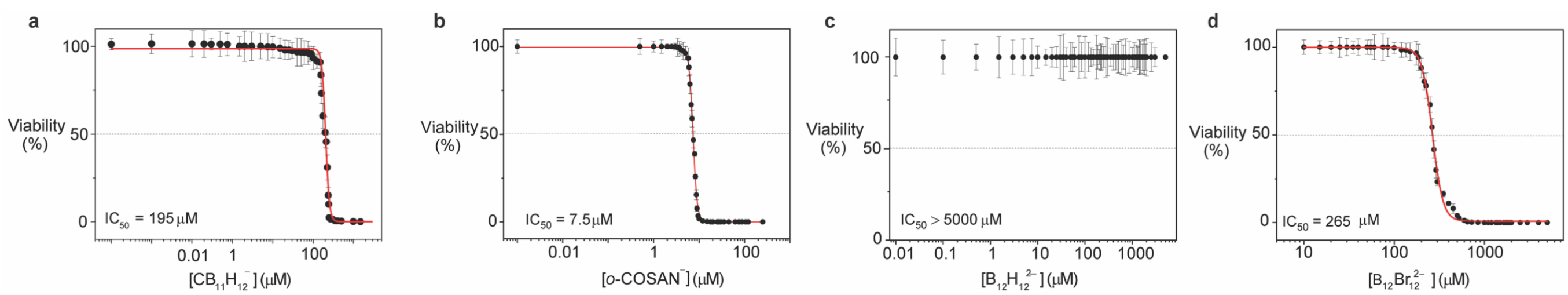

Figure S20: Viability of CHO-K1 cells in the presence of **a** $CB_{11}H_{12}^{-}$, **b** *o*-COSAN^{-}, **c** $B_{12}H_{12}^{-}$ and **d** $B_{12}Br_{12}^{-}$ estimated by the resazurin assay after 24 hours incubation. 10% (SD) error for IC_{50}.

10. References

[1] W. Knoth, 1-B9H9CH-and B11H11CH. *J. Am. Chem. Soc.* **1967**, *89*, 1274-1275.

[2] B. Grüner, Z. Janoušek, B. T. King, J. N. Woodford, C. H. Wang, V. Všetečka, J. Michl, Synthesis of 12-Substituted 1-Carba-closo-dodecaborate Anions and First Hyperpolarizability of the 12-C7H6+-CB11H11- Ylide. *J. Am. Chem. Soc.* **1999**, *121*, 3122-3126.

[3] B. Gruner, I. Cisarova, J. Caslavsky, B. Bonnetot, D. Cornu, Synthesis of 12-hydroxy and 12-dioxane derivatives of the closo-1-carbadodecaborate(1-) ion. Variations on the Plesek's cobalt bis(dicarbollide) pattern. *Collect. Czech. Chem. Commun.* **2002**, *67*, 953-964.

[4] J. Plešek, T. Jelínek, E. Drdáková, S. Heřmánek, B. Štíbr, A convenient preparation of 1-CB 11 H 12-and its C-amino derivatives. *Collect. Czech. Chem. Commun.* **1984**, *49*, 1559-1562.

[5] H. Mach, C. R. Middaugh, R. V. Lewis, Statistical determination of the average values of the extinction coefficients of tryptophan and tyrosine in native proteins. *Anal. Biochem.* **1992**, *200*, 74-80.

[6] https://www.aatbio.com/products/5-tamra-5-carboxytetramethylrhodamine-cas-91809-66-4?unit=363. (accessed 7 May 2026).

[7] J. C. M. Stewart, Colorimetric determination of phospholipids with ammonium ferrothiocyanate. *Anal. Biochem.* **1980**, *104*, 10-14.

[8] M. Nishihara, F. Perret, T. Takeuchi, S. Futaki, A. N. Lazar, A. W. Coleman, N. Sakai, S. Matile, Arginine magic with new counterions up the sleeve. *Org. Biomol. Chem.* **2005**, *3*, 1659-1669.

[9] A. D. Becke, Density-functional thermochemistry. III. The role of exact exchange. *J. Chem. Phys* **1993**, *98*, 5648-5652.

[10] R. Krishnan, J. S. Binkley, R. Seeger, J. A. Pople, Self-consistent molecular orbital methods. XX. A basis set for correlated wave functions. *J. Chem. Phys* **1980**, *72*, 650-654.

[11] A. Barba-Bon, A. El Haitami, C. Pasquier, I. Nikšić-Franjić, O. Diat, P. Bauduin, S. Cantin, W. M. Nau, Boron Cluster Anions Dissolve En Masse in Lipids Causing Membrane Expansion and Thinning. *Angew. Chem. Int. Ed.* **2024**, *63*, e202412834.

[12] Y. Yang, M. N. Weaver, K. M. Merz Jr, Assessment of the "6-31+ G**+ LANL2DZ" mixed basis set coupled with density functional theory methods and the effective core potential: prediction of heats of formation and ionization potentials for first-row-transition-metal complexes. *The Journal of Physical Chemistry A* **2009**, *113*, 9843-9851.

[13] S. Grimme, J. Antony, S. Ehrlich, H. Krieg, A consistent and accurate ab initio parametrization of density functional dispersion correction (DFT-D) for the 94 elements H-Pu. *J. Chem. Phys.* **2010**, *132*, 154104.

[14] Y. Zhao, D. G. Truhlar, The M06 suite of density functionals for main group thermochemistry, thermochemical kinetics, noncovalent interactions, excited states, and transition elements: two new functionals and systematic testing of four M06-class functionals and 12 other functionals. *Theor. Chem. Acc.* **2008**, *120*, 215-241.

[15] A. V. Marenich, C. J. Cramer, D. G. Truhlar, Universal solvation model based on solute electron density and on a continuum model of the solvent defined by the bulk dielectric constant and atomic surface tensions. *J. Phys. Chem. B* **2009**, *113*, 6378-6396.

[16] R. C. Gaussian 16, M. J. Frisch, G. W. Trucks, H. B. Schlegel, G. E. Scuseria, M. A. Robb, J. R. Cheeseman, G. Scalmani, V. Barone, G. A. Petersson, H. Nakatsuji, X. Li, M. Caricato, A. V. Marenich, J. Bloino, B. G. Janesko, R. Gomperts, B. Mennucci, H. P. Hratchian, J. V. Ortiz, A. F. Izmaylov, J. L. Sonnenberg, D. Williams-Young, F. Ding, F. Lipparini, F. Egidi, J. Goings, B. Peng, A. Petrone, T. Henderson, D. Ranasinghe, V. G. Zakrzewski, J. Gao, N. Rega, G. Zheng, W. Liang, M. Hada, M. Ehara, K. Toyota, R. Fukuda, J. Hasegawa, M. Ishida, T. Nakajima, Y. Honda, O. Kitao, H. Nakai, T. Vreven, K. Throssell, J. A. Montgomery, Jr., J. E. Peralta, F. Ogliaro, M. J. Bearpark, J. J. Heyd, E. N. Brothers, K. N. Kudin, V. N. Staroverov, T. A. Keith, R. Kobayashi, J. Normand, K. Raghavachari, A. P. Rendell, J. C. Burant, S. S. Iyengar, J. Tomasi, M. Cossi, J. M. Millam, M. Klene, C. Adamo, R. Cammi, J. W. Ochterski, R. L. Martin, K. Morokuma, O. Farkas, J. B. Foresman, and D. J. Fox,, Gaussian, Inc., Wallingford CT, **2016**.

[17] I. Hypercube, Gainesville, FL: Hypercube, Inc, **2007**.

[18] PyMOL Molecular Graphics System, Version 3.0 Schrödinger, LLC.
